# Genomic and Proteomic Characterizations of *Sfin-1*, a Novel Lytic Phage Infecting Multidrug-Resistant *Shigella* spp. and *Escherichia coli* C

**DOI:** 10.3389/fmicb.2019.01876

**Published:** 2019-08-22

**Authors:** SK Tousif Ahamed, Banibrata Roy, Utpal Basu, Shanta Dutta, A. N. Ghosh, Boudhayan Bandyopadhyay, Nabanita Giri

**Affiliations:** ^1^Department of Microbiology, Acharya Prafulla Chandra College, Kolkata, India; ^2^Department of Molecular Biology and Biotechnology, University of Kalyani, Kalyani, India; ^3^Division of Bacteriology, National Institute of Cholera and Enteric Diseases, Kolkata, India; ^4^School of Advanced Sciences and Languages, VIT Bhopal University, Bhopal, India

**Keywords:** bacteriophage, *Shigella* spp., phage therapy, genome sequencing, large terminase, LC-MS/MS

## Abstract

Shigellosis is a public health threat in developed as well as developing countries like “India.” While antibiotic therapy is the mainstay of treatment for shigellosis, current emergence of multidrug-resistant strains of *Shigella* spp. has posed the problem more challenging. Lytic bacteriophages which destroy antibiotic resistant *Shigella* spp. have great potential in this context and hence their identification and detailed characterization is necessary. In this study we presented the isolation and a detailed characterization of a novel bacteriophage *Sfin-1*, which shows potent lytic activity against multidrug-resistant isolates of *Shigella flexneri*, *Shigella dysenteriae*, *Shigella sonnei* obtained from clinical specimens from shigellosis patients. It is also active against *Escherichia coli* C. The purified phage is lytic in nature, exhibited absorption within 5–10 min, a latent period of 5–20 min and burst size of ∼28 to ∼146 PFU/cell. The isolated phage shows stability in a broad pH range and survives an hour at 50°C. Genome sequencing and phylogenetic analyses showed that *Sfin-1* is a novel bacteriophage, which is very closely related to T1-like phages (89.59% identity with Escherichia virus T1). *In silico* analysis indicates that *Sfin-1* genome consists of double stranded linear DNA of 50,403 bp (GC content of 45.2%) encoding 82 potential coding sequences, several potential promoters and transcriptional terminators. Under electron microscopy, *Sfin-1* shows morphology characteristics of the family *Siphoviridae* with an isometric head (61 nm) and a non-contractile tail (155 nm). This is most likely the first report of a lytic bacteriophage that is active against three of the most virulent multidrug-resistant *Shigella* species and therefore might have a potential role in phage therapy of patients infected with these organisms.

## Introduction

Instances of Shigellosis infection were estimated to be about 170 million annually during the end of last century with about 1 million reported deaths in the developing countries. Although this number has decreased, shigellosis still presents itself as one of the most important pandemic diseases in the world (Sur et al., 2004).

Shigellosis is caused by the bacteria of the genus *Shigella* having four pathogenic serogroups (*Shigella dysenteriae*, *Shigella flexneri*, *Shigella sonnei*, and *Shigella boydii*). Infection is common by feco-oral route due to the intake of contaminated water and food (Baird-Parker, 1994). Although WHO recommends antibiotics like azithromycin, ciprofloxacin [a fluoroquinolone (FQ)] or one of the three second-line antibiotics, pivmecillinam, and ceftriaxone (a third-generation cephalosporin) for the shigellosis treatment, extensive use of these antibiotics has contributed to the emergence of FQ and multidrug-resistant *Shigella* species in several countries (Sivapalasingam et al., 2006; Von Seidlein et al., 2006; Yismaw et al., 2008; Nandy et al., 2010; Tariq et al., 2012; Muthuirulandi Sethuvel et al., 2017; Puzari et al., 2017). Thus, recurrent changes in antimicrobial resistance profile of *Shigella* isolates poses complications in recommending standard drugs for effective treatment of the disease. Currently, the most predominant species in endemic regions was *S. flexneri* followed by *S. sonnei*, *S. boydii*, and *S. dysenteriae* which may be responsible for causing diarrheal outbreak (Sengupta et al., 1990; Nandy et al., 2010).

Bacteriophages are specific bacterial viruses that at first attach to and then destroy their hosts through phage genome replication and bacterial lysis. These characteristics of phages hint at their applications as therapeutic agents against bacterial infections of humans and animals. The fact that, unlike antibiotics, phages can destroy target bacteria specifically without killing the normal microflora. It is crucial for the application of phages to inhibit specific bacterial growth, which can be a natural, non-toxic and active substitute for antibiotic therapy (O’flaherty et al., 2005; Gupta and Prasad, 2011). In view of the enormous rise in antibiotic resistance in several clinically significant bacterial species, use of lytic phages is gaining more and more attention as a therapeutic alternative in the place of antibiotics against infectious diseases.

However, numerous challenges have to face during application of phage therapy in human diseases due to the limited available information regarding the interaction between phage and the host (Housby and Mann, 2009). Again, for the application of phage therapy, it is necessary to isolate new phages that are yet to be cultivated, active against circulating strains and to determine their physiological and genomic characters in detail for assessing their suitability.

Several bacteriophages against *Shigella* spp. have been reported. Temperate bacteriophages such as SfI(38,389 bp), SfII(41,475 bp), Sf6(39,043 bp), SfIV(39,758 bp), SfV(37,074 bp), and SfX(37,355 bp) (Mavris et al., 1997; Guan et al., 1999; Allison et al., 2002; Casjens et al., 2004; Jakhetia et al., 2013; Sun et al., 2013) that are responsible in serotype conversion of *S. flexneri* have been reported. The lytic Shigella phages SF9, SP18 and ØSboM-AG3 are specific against *S. dysenteriae*, *S. sonnei*, and *S. boydii*, respectively. One broad spectrum Shigella phage pSf-1 was reported few years back that was specific against *S. flexneri*, *S. sonnei*, and *S. boydii*; another recently discovered phage vB_SsoS-ISF002 can infect both *S. flexneri* and *S. sonnei* but broad spectrum lytic phage that infects both *S. flexneri* and *S. dysenteriae* has not been reported yet (Allison and Verma, 2000; Faruque et al., 2003; Kim et al., 2010; Anany et al., 2011; Jun et al., 2013; Shahin et al., 2018).

Generally phages acquire and contribute genes, not only to other phage genomes but also to bacterial genomes and thus these are powerful factors in the evolution, physiology, and pathogenicity of the host bacteria. Here, we report the isolation and detailed characterization of a novel bacteriophage *Sfin-1*, which shows strong lytic activity against multidrug-resistant isolates of *S. flexneri*, *S. dysenteriae*, and *S. sonnei.* Discovery of new phage against *Shigella* spp. and their genomics would not only help to develop phage based therapy against the shigellosis, but also to understand the evolution strategy, the mosaic architecture of phages and the involvement of genes(s) if any, in host pathogenesis.

## Materials and Methods

### Bacterial Strains and Antimicrobial Resistance Tests

This study included a total number of 40 multidrug-resistant clinical isolates of *S. flexneri*, *S. dysenteriae*, *S. boydii*, *S. sonnei*, *Salmonella enterica serovar* Typhi, various *Escherichia coli* strains like K12, AG100, XL1 Blue, and *E. coli* C. All *Shigella* and *Salmonella* species were isolated from patient’s stool samples at Bacteriology Division of National Institute of Cholera and Enteric Diseases (NICED), “Kolkata” and reported thereafter (Nandy et al., 2010; Dutta et al., 2014). They were then grown in nutrient broth medium at 37°C for 24 h for further tests adhering to the biosecurity and institutional safety procedures under Biosafety Level II (BSL II).

### Isolation and Purification of Bacteriophages

Water sample was collected from the Ganga River, near Serampore, Hooghly district, about 25 km from Kolkata, in the state of West Bengal, India. After the removal of particulate matters with filter paper (Whatman 1), the water sample was mixed with *S. flexneri* 2a strains, 10% (w/v) peptone and it was incubated at 37°C for 24 h to enrich the concentration of bacteriophages. The enriched culture was then mixed with 1% (w/v) chloroforms and was shaken well to remove the bacterial debris. The mixture was centrifuged and supernatant was filtered through 0.22 μm pore membrane (Millipore, United States). This supernatant was inoculated (10 μL) as spot on the soft agar plate mixed with *S. flexneri* 2a strains. Clear zone around the spot indicated the presence of specific bacteriophage in the water sample against *S. flexneri* 2a. Similarly, other isolated serotypes of *Shigella* spp. were also checked for determination of phage specificity.

Collected water sample was then subsequently used for plaque assay; 100 μL of filtrate and 200 μL *S. flexneri* 2a culture (OD600 = 0.3) was mixed with 3.5 mL soft agar (0.9%) and plated onto LB hard agar (1.8%) plate. This plate was incubated in 37°C for 24 h. Clear individual plaques formed on the plate were transferred to another *S. flexneri* 2a plate. A single plaque was transferred into 500 μL phage dilution medium (0.1% tryptone, 0.85% sodium chloride) and stored at 4°C for 24 h. The suspended phage solution was then allowed for another round of plaque assay. Thus a single plaque was transferred at least three times to purify the bacteriophage. After that, phage dilutions and plaque assay was performed to get confluent lysis plates. The soft agar layer was then scrapped out, suspended in cold phage dilution medium and kept on ice for 3 h. It was then centrifuged at 5,000 × *g* and the supernatant was collected. The phage lysate prepared by this procedure was then pelleted at 68,000 × *g* for 2 h at 4°C in an ultracentrifuge since phage precipitates produced thus has more titer values. For further purification, cesium chloride (CsCl) density gradient centrifugation was done (ρ = 1.3, 1.5, 1.7 g/mL) at 100,000 × *g* for 3 h at 4°C and the phage band obtained between 1.7 and 1.5 g/mL was collected. The phage particles were recovered from the band and were dialyzed against TM buffer (50 mM Tris–Cl, pH 8.0 containing 10 mM MgSO_4_) and stored at 4°C for further studies (Uchiyama et al., 2008).

### Host Range

The strains to be tested were grown overnight in nutrient broth 3.5 mL of the molten soft agar (0.7% w/v) was mixed with 100 μL of the bacterial cell suspension and this mixture was overlaid onto the surface of solid basal LB Agar (1.5% w/v). 10 μL (about 1.0 × 10^10^ PFU/mL) of the phage suspension was spotted on the plate and it was then incubated at 37°C, overnight. Bacterial sensitivity to a bacteriophage was determined by bacterial lysis at the spot where the phage suspension was inoculated. Each test was repeated three times. According to the degrees of clarity, the spots were differentiated into two categories: clear (+), and no reaction (−) (Bennish et al., 1990; Chang et al., 2005).

### Thermal and pH Stability

For thermal stability testing, 16 × 10^12^ *Sfin-1* phage particles were incubated at 4, 37, 50, 60, 70, 80, 90°C in 1 mL and for each temperature aliquots (100 μL) were taken after 5, 15, 40, 60 min to be titered by the double layered agar plate method against *S. flexineri* 2a. For pH stability testing, 14 × 10^10^ *Sfin-1* phage particles were kept in 1 mL of TM buffers (50 mM Tris, 10 mM MgSO_4_) at pH range of 2–12 (adjusted with HCl or NaOH for acidic or alkaline range, respectively) for 1 h at 37°C and then aliquots (100 μL) from each pH were titered by the double-layered agar plate method against *S. flexineri* 2a (Yoon et al., 2007; Ma and Lu, 2008).

### Bacterial Challenge Test

Minimum inhibitory concentrations (MICs) of different antibiotics for three clinical *Shigella* isolates were determined. According to the CLSI guidelines (CLSI, 2016) the isolates were multi drug resistant. Bacteriolytic activity of phage *Sfin-1* was determined as described by Wang et al. (2016) with some modifications. Cells of *S. flexneri* 2a *(1A)* (strain ID BCH5722, Table 1) and *S. dysenteriae*1 *(1A)* (strain ID BCH5762, Table 1) were allowed to grow in presence of ampicillin (32 μg/mL), chloramphenicol (32 μg/mL), tetracycline (16 μg/mL), cotrimoxazole (25 μg/mL), nalidixic acid (32 μg/mL), ciprofloxacin (4 μg/mL), norfloxacin (16 μg/mL), and ofloxacin (8 μg/mL), whereas *S. sonnei* (strain ID BCH7084, Table 1) was grown in presence of tetracycline (16 μg/mL), cotrimoxazole (25 μg/mL), and nalidixic acid (32 μg/mL). 20 mL of cultures (OD600 = 0.3) were harvested by centrifugation and resuspended in fresh 1 mL of LB. Thereafter phage *Sfin-1* was added at an multiplicity of infection (MOI) of 0.1, 0.01, and 0.001, allowed for adsorption for 5 min (*S. flexneri 2a* and *S. dysenteriae1*) or 10 min (*S. sonnei*) at 37°C. Each mixture was then transferred to 20 mL of fresh LB. Aliquots were taken at specific time intervals for 5 h and the number of bacterial cells was monitored by spread plate method. As negative control bacterial cultures were inoculated with phage dilution medium and respective antibiotics.

**TABLE 1:** Susceptibility test for various clinically isolated antibiotic resistant strains to the phage *Sfin-1*, isolated from river Ganges water samples Kolkata, India.

**Sl. no**	**Strain ID**	**Bacterial isolates with different serotypes**	**Antimicrobial resistance profile by disc diffusion method**	**Lysis by *Sfin-1***
1	BCH5722	*Shigella flexneri 2a (1A)*	ACTQNaCipNorOfx	+
2	BCH4025	*Shigella flexneri 2a (2A)*	ACQ	+
3	BCH3651	*Shigella flexneri 2a (3A)*	ACTQ	+
4	BCH3557	*Shigella flexneri 2a (4A)*	CTQNa	+
5	BCH7151	*Shigella flexneri 2a (5A)*	ACTQNaCipNorOfx	+
6	BCH5762	*Shigella dysenteriae 1(1A)*	ACTQNaCipNorOfx	+
7	BCH5848	*Shigella dysenteriae 1(2A)*	ACTQNaCipNorOfx	+
8	BCH5859	*Shigella dysenteriae 1(3A)*	ACTQNaCipNorOfx	+
9	BCH5912	*Shigella dysenteriae 1(4A)*	ACTQNaCipNorOfx	+
10	BCH5946	*Shigella dysenteriae 1(5A)*	ACTQNaCipNorOfx	+
11	BCH7084	*Shigella sonnei (1)*	TQNa	+
12	BCH7264	*Shigella sonnei (2)*	TQNa	+
13	BCH3143	*Shigella boydii (1)*	TQNa	−
14	BCH4324	*Shigella boydii (2)*	TQNa	−
15	BCR62	*Salmonella enterica serovar* Typhi *(1)*	NaCipNorAzm	−
16	BCR43	*Salmonella enterica serovar* Typhi *(2)*	NaAzm	−
17		*Escherichia coli K12*		−
18		*Escherichia coli* C		+
19		*Escherichia coli AG100*		−
20		*XL1 Blue*		−

*A, ampicillin; C, chloramphenicol; T, tetracycline; Q, cotrimoxazole; Na, nalidixic acid; Cip, ciprofloxacin; Nor, norfloxacin; Ofx, ofloxacin; Azm, azithromycin.*

### One Step Growth Curve

One step growth experiments were carried out by a method described elsewhere (Chang et al., 2005; Malek et al., 2009) with modification. Briefly, *Shigella* spp. (*S. flexneri* 2a *(1A)*, *S. dysenteriae* 1 *(1A)*, *S. sonnei*) were grown in LB medium at 37°C. Then 20 mL of *Shigella* culture (OD600 = 0.3) was harvested by centrifugation (5,000 × *g*, 4°C, 10 min). The pellet was resuspended in 1 mL of fresh LB and phage *Sfin-1* was added to Shigella culture at a MOI of 0.01. The mixture was incubated for maximum adsorption (5 min for *S. flexneri* 2a and *S. dysenteriae* 1, 7 min for *S. sonnei*) at 37°C followed by 10^4^-fold of dilution at final volume of 10 mL. During incubation at 37°C, 100 μL of aliquots were taken at different time intervals up to 100 min, mixed with 200 μL of *S. flexineri 2a* culture, plated on double-layered agar for phage titration. Three independent experiments were run for each *Shigella* spp. Burst size was determined as a ratio of the average bacteriophage particles produced after the burst and the average number of phage particles adsorbed.

### Genome Analysis of Phage *Sfin-1*

Bacteriophage DNA was isolated from purified phages (procedure described above), using the phage DNA isolation kit (Norgen, Canada) according to the manufacturer’s instruction with modifications (Berg et al., 2016). Whole genome sequencing of *Sfin-1* was performed by ION Xpress (S5-00205) version 5.0.4. The count of read was 67,228 with the average length of 322 bp per read. The sequence data could be assembled into a single contig of 50,403 bp using SPAdes 3.8.0 (Bankevich et al., 2012). The nucleotide sequence of the genome of *Sfin-1* was submitted at GenBank under accession number MF468274. The nucleotide sequences of the phage *Sfin-1* were auto annotated by GeneMarkS^1^ (Ver 3.26) (Besemer et al., 2001) and the function of the proteins encoded by the coding sequences (CDSs) were predicted based on BLASTp program and conserved domain search^2^. The probable replication origin of *Sfin-1* was predicted by GenSkew program^3^. Putative promoter regions were predicted using Neural Network Promoter Prediction tool of the Berkeley Drosophila Genome Project^4^ (minimum promoter score: 0.9). Rho-independent transcription terminators were identified using ARNOLD terminator finding program (Lesnik et al., 2001). tRNA scan–SE search program^5^ was used to detect Putative tRNAs, if any (Lowe and Chan, 2016). Whole genome comparisons were made with Mauve^6^. Sequences of tail fiber proteins were compared by using the online Protein Predict Tool (Yachdav et al., 2014).

### Electron Microscopy

The phage suspension (about 1 × 10^12^ PFU/mL) was negatively stained with 2% (w/v) uranyl-acetate and then examined under a FEI Tecnai 12 Bio Twin Transmission Electron Microscope at an operating voltage of 200 kV.

### Identification of Proteins Associated With *Sfin-1* Virions

A total of 100 μL (1 × 10^18^ PFU/mL) purified phage was diluted with 50 mM NH_4_HCO_3_. It was then treated with 100 mM DTT at 95°C for 1 h followed by 250 mM iodoacetamide at room temperature in dark for 45 min. The sample was then digested with Trypsin and incubated overnight at 37°C. The peptides were extracted in 0.1% formic acid and incubated at 37°C for 45 min. The solution thus prepared was centrifuged at 10,000 × *g* and the supernatant was vacuum dried and dissolved in 20 μL of 0.1% formic acid in water. 10 μL of it was subjected to ACQUITY UPLC BEH C18 column (Waters, United Kingdom) for separation of peptides; the peptides separated on the column were directed to Waters Synapt G2 Q-TOF instrument (Waters, United Kingdom) for MS and MS/MS analysis. The raw data was processed using MassLynx 4.1 WATERS. The individual peptides MS/MS spectra were matched to the database sequence for protein identification on PLGS software, Waters. The obtained spectrometry information was analyzed with PLGS software 3.0.2 (Waters, United Kingdom) using the National Centre for Biotechnology Information (NCBI) non-redundant database and the specific database created in this study based on the predicted CDSs of phage *Sfin-1*. The important parameter settings for the PLGS analysis were as follows: peptide mass tolerance (ppm), 50; fragment mass tolerance (ppm), 100; maximal missed cleavages, 2.

### Determination of the Bacteriophage Genome Ends

The identification of phage packaging strategies and genome ends of a bacteriophage can be obtained by comparative analysis of phylogenetic relationships of amino acid sequences of terminase large subunit of phage with the other phages of known packaging strategies (Wittmann et al., 2014; Amarillas et al., 2017). The phylogenetic tree was thus reconstructed using the large terminase amino acid sequence of the phages and the relationships among the phage *Sfin-1* and with the other phages were analyzed.

The predicted amino acid sequences of the large terminase subunits genes of the phages were collected from NCBI and were used for phylogenetic analysis. The bacteriophages involved into this study have been molecularly analyzed and contains well characterized ds DNA bacteriophages with different types of packaging strategies dependent on terminase actions (headful, 5′-extended cos ends, 3′-extended cos ends and direct terminal repeats). All the sequences were aligned using ClustalW in MEGA7 with default parameters. Phylogenetic tree was built using the neighbor-joining method and phylogenies were determined by bootstrap analysis of 1,000 replicates in MEGA 7.0 version (Filipski et al., 2014).

Additionally the genome ends were determined as described by Amarillas and Leon-Felix (Amarillas et al., 2017). To detect the presence of circularly permutated terminally redundant genome ends, approximately 1 μg phage DNA was digested with specific restriction enzymes (*Bgl*II, *Mlu*I) according to the manufacturer’s guidelines (NEB, United States). After 2 h of incubation at 37°C the digests were heated to 80°C for 15 min and then cooled fast in ice or slow at room temperature and allowed to run on agarose gel (0.8% w/v) in TAE electrophoresis buffer. The gel was stained with ethidium bromide and visualized with UV illumination. GeneRuler 1 kb Plus DNA Ladder (Thermo Fisher Scientific, United States) was used as DNA molecular weight marker.

### Phage Receptor Identification

The receptor properties of *Sfin-1* were determined as describe previously with some modifications (Kiljunen et al., 2011). To check the effect of proteinase K on adsorption of phage *Sfin-1*, *S. flexneri 2a*, *S. dysenteriae 1*, and *S. sonnei* cultures (OD600 = 0.3) were treated with proteinase K (250 mg/mL, SRL) at 55°C for 2 h, washed and allowed for adsorption assay at an MOI of 0.0001 as described above. In order to study whether periodate can inhibit *Sfin-1* and hosts interaction, the following experiment was performed. *S. flexneri 2a*, *S. dysenteriae1*, and *S. sonnei* cultures were centrifuged at 5,000 × *g* for 5 min, bacterial pellets were resuspended into sodium acetate (50 mM, pH 5.2) in presence or absence of 200 mM NaIO_4_ and incubated for 2 h (protected from light). After incubation the cells were washed and performed adsorption assay.

To ensure that the possible effect was not due to the incubation of host cells at 55°C, a control experiment without proteinase K was also performed. LB medium was used as a non-absorbing control for both assays. The phage titer in the control supernatant was set to 100%.

### Statistical Analysis

For the thermal stability assays, the difference in the titer values taken between 0 and 60 min were calculated for each temperature. Then the titer value difference for each temperature was compared to that of 4°C using Student’s *t*-test. Two way ANOVA test was performed to analyze the data of Bacterial challenge test. Student’s *t*-tests were performed to analyze the data of phage receptor identification. All statistical analysis were performed by software GraphPad Prism 7.0.

## Results and Discussion

### Isolation of Bacteriophage

**FIGURE 1:**
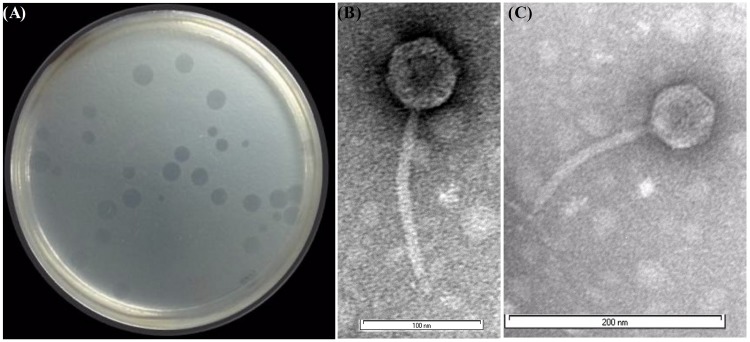
*Shgella* spp. specific phage *Sfin-1* and its morphology. **(A)** Plaques of *Sfin-1* in the lawn of *Shigella flexneri 2a*. The phage particles were prepared, negatively stained and examined by electron microscope as described in “Materials and Methods” section. **(B,C)** The electron micrograph presented as broad view of the phage in 100 and 200 nm scale, respectively.

Several environmental water samples from ponds, creeks, streams, and canal ways were collected and the presence of phage that infects *Shigella* spp. was tested according to the method described in “Materials and Methods” section. The water sample from the River Ganges, Kolkata (located near Serampore, Hooghly of West Bengal, India) was found to contain phage, designated as *Sfin-1* that could grow in antibiotic resistant various strains of *Shigella* spp. and resulted in clear lytic plaques with size ranging from 1.5 to 2.0 mm in diameter and well defined boundaries in the lawn of the bacteria (Figure 1A). Purification of the virion was done by CsCl density gradient ultracentrifugation after plaque purification and phage propagation as described in “Materials and Methods” section. The transmission electron microscopy (TEM) photos and the genome organization (described below) suggest that only one type of phage was present into the purified sample. If more than one bacteriophage were present there, then more genes of specific phage proteins such as tape measure protein and large terminase subunit would have been expected.

### Broad Host Range

The ability to lyse various pathogenic *Shigella* spp. of newly isolated phage was analyzed by the spot test. The strains belonging to the Shiga toxin producing clinical isolates of *S. flexneri*, *S. dysenteriae*, *S. boydii*, *S. Sonnei* were used. Other enteropathogens like *Salmonella typhi* and various *E. coli* strains like K12, AG100, XL1 Blue and *E. coli* C. were also checked. The *Shigella* serovars are resistant to multiple antibiotics (amoxicillin, chloramphenicol, tetracycline, ciprofloxacin, norfloxacin, nalidixic acid, cotrimoxazole, ofloxacin, and azithromycin) (Amezquita-Lopez et al., 2014) (Table 1). Phage suspensions produced clear zones of lysis in case of various serotypes of *S. flexneri*, *S. dysenteriae*, *S. sonnei*, and *E. coli* C., but no reaction was observed for other species.

Our host range studies suggest that *Sfin-1* is a newly isolated phage with a broad lytic spectrum. The wide host range infectivity against various *Shigella* isolates shows that *Sfin-1* is a polyvalent phage on *Shigella*, known human and animal pathogen and *E. coli* C. Although phages are commonly very specific infecting only a single species of bacteria, some polyvalent phages have also been described (Hamdi et al., 2017).

From the Ganges River that flows through the city of Kolkata, we isolated the phage *Sfin-1* near Serampore, Hooghly. Since *Shigella* spp. is communicated to human by fecal-oral route, the isolation of phage *Sfin-1* indicates fecal contamination of the river. This lytic phage is active against a broad host range of antibiotic resistant strains of *S. flexneri*, *S. dysenteriae*, and *S. sonnei.* This is the first report of bacteriophage that is lytic against three pathogenic *Shigella* spp. and *E. coli* C. Therefore, it can be an excellent choice for phage therapy against antibiotic resistant Shigellosis.

### Phage Morphology

The phage *Sfin-1* which is specific against various antibiotic resistant *Shigella* spp. was purified and subjected to TEM. The TEM study suggested that this phage has an isometric head (61 nm in diameter) and a non-contractile tail (approximately 155 nm) with which a basal tuft is attached (Figures 1B,C). The mature phage does not have neck, base plate, spikes, or fiber. According to the guidelines of the International Committee on Taxonomy of Viruses (Fauquet and Fargette, 2005), which is based on virion morphological features, the phage *Sfin-1* was assigned to the family *Siphoviridae* in the order *Caudovirale*s. More than 95% of the phages reported so far grouped into the order Caudovirales (tailed phages). According to the classification system of Ackermann (1998), most (60%) of the bacteriophages belong to the family *Siphoviridae* with flexible and long tails. So the phage *Sfin-1* belongs to this taxonomic classification.

### Bacterial Challenge Test

**FIGURE 2:**
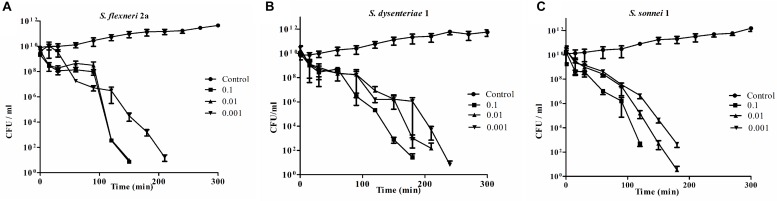
Bacterial challenge test of phage *Sfin-1* on different clinical isolates of *Shigella* spp. Clinically isolated species of **(A)** *Shigella flexneri 2a*, **(B)** *Shigella dysenteriae* 1, and **(C)** *Shigella sonnei* were grown (OD600 = 0.3) in 20 mL LB in presence of several antibiotics as described in “Materials and Methods” section, hervested by centrifugation, resuspended in 1 mL LB medium and infected with *Sfin-1* at an MOI of 0.1, 0.01, and 0.001. After adsorption, the cultures were diluted 21 fold in LB and incubated for 5 h with shaking at 37°C. At different time intervals, viability of *Shigella* spp. was determined by spread plate method. As negative control *Shigell* spp. were grown in absence of *Sfin-1* in presence of antibiotics. Two way ANOVA indicated significant difference between control and *Sfin-1* infected sets (*P* < 0.0001, *n* = 3).

With the addition of phage *Sfin-1* at an MOI of 0.001, 0.01, and 0.1 to mid-exponential phase cells (OD600 = 0.3) in *in vitro* culture conditions, challenge tests were performed to investigate the ability of phage *Sfin-1* to lyse multidrug-resistant *S. flexneri 2a (1A)* (Strain Id BCH5722, Table 1) in presence of antibiotics ampicillin, chloramphenicol, tetracycline, cotrimoxazole, ciprofloxacin, norfloxacinand ofloxacin (Figure 2A). In each case, control experiment was performed where bacterial cells were grown only in presence of respective antibiotics and phage suspension buffer without phage particles. The viability of bacterial cells was significantly decreased when infected with MOI of 0.1, 0.01, and 0.001. The two way ANOVA tests for multiple comparisons showed that the mean differences between the cell lysis data for all three MOIs and control are highly significant (*P* < 0.0001). Within one and half hours after phage addition, cells were started to decrease rapidly and complete lysis occurred within two and half hours in case of MOI of 0.1 and 0.01. But in case of 0.001, complete lysis occurs after 3 h. Similar results were observed for *S. dysenteriae1 (1A)* (Strain ID BCH5762, Table 1) in presence of the same antibiotics as mentioned above (Figure 2B) and *S. sonnei* (Strain ID BCH7084) cells in presence of tetracycline, cotrimoxazole, and nalidixic acid (Figure 2C). The *in vitro* challenge tests established that the phage *Sfin-1* could be used to inactivate the multidrug-resistant pathogenic strains of *Shigella* and therefore, it could be useful as biocontrol agent. The efficacy of this phage in controlling *Shigella* infection however has to be determined by *in vivo* studies. It is important to mention that a host population known as bacterial insensitive mutants (BIMs) that can resist lysis may emerge and grow again long after phage treatment (Yordpratum et al., 2011; Yamaki et al., 2014; Amarillas et al., 2017). Use of phage cocktail with more than one phage that follows different infection mechanisms may solve this problem (Yamaki et al., 2014).

So the result of host cell lysis caused by phage *Sfin-1* showed that MOI is directly related to cell death. If higher number of phages are applied on cells, destabilization of the outer membrane occurs, which in turn causes cell lysis. Since it is not the result of phage replication and release, it is called “lysis from without” (Brown and Bidle, 2014; Amarillas et al., 2017). In Supplementary Table S1 phage *Sfin-1* is compared with other 44 Shigella phages so far reported in the NCBI database. Only 26% of the isolated Shigella phages have been characterized till date.

### Infectivity of *Sfin-1*

**FIGURE 3:**
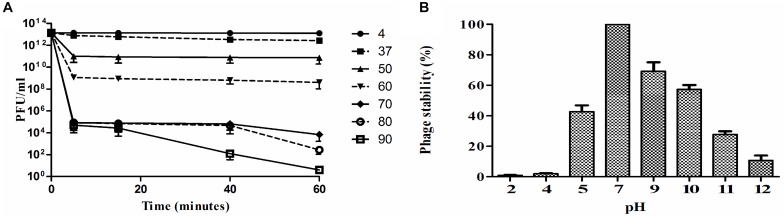
Stability of phage *Sfin-1* in wide temperature and pH range. **(A)** Thermal stability of phage *Sfin-1* at various temperatures as indicated. *Sfin-1* phage particles (16 × 10^12^) were incubated at different temperatures in 1 mL and for each temperature the number of infectious phage particles was determined from 100 μL aliquots from various time points by plaque assay against *S. flexineri* 2a. Result was plotted as mean ± SD (*n* = 3). **(B)** pH stability of phage *Sfin-1*. In 1 mL of TM buffer having different pH *Sfin-1* phage particles (14 × 10^10^) were incubated at 37°C for 1 h and the number of infectious phage particles from each sample was determined with 100 μL aliquots by plaque assay against *S. flexineri* 2a. Result was plotted as mean ± SD (*n* = 3).

Thermal stability test was carried out with *Sfin-1* at pH 7.0 in order to investigate the heat resistant capability of the phage. The activity of phage *Sfin-1* remained moderately same when warmed at 37°C or 50°C for 5 min. Activity decreased significantly into 0.1–0.01% when incubated at 60°C or 70°C for 5 min (*P* < 0.005). When heated at 80°C or 90°C for 5 min only 0.001% activity was retained (*P* < 0.005). Most of the phages remained active even after 60 min incubation at 37°C or 50°C, whereas only 0.1 and 0.01% phages were active after 60 min incubation at 60 and 70°C, respectively (*P* < 0.005), the phage activity remarkably decreased at 80°C or 90°C after 60 min incubation (*P* < 0.0005). This result suggests that the phage *Sfin-1* is moderately stable toward heat stress at both 37 and 50°C (Figure 3A).

The *Shigella* infection commonly happens in intestine where the pH is somewhat acidic. In order to control *Shigella* with *Sfin-1* in human intestine, it is therefore, essential to know its pH stability. Highest activity was observed after 1 h incubation at pH 7.0 at 37°C, while reduction of activity was observed at different pH. Around 42.7% or 10.8% recovery of infectious phage *Sfin-1* was found at pH 5.0 and 12.0, respectively. This result suggested that extreme pH as well as lower pH though affect the phage stability but a remarkable fraction of *Sfin-1* remained active (Figure 3B). Rapid absorption, moderate thermal and pH stability therefore indicate that this phage may be applied for therapeutic purpose. However, in order to achieve therapeutic efficacy the phages must be delivered to the small intestine through encapsulation otherwise it may not survive at low gastric pH (Vinner et al., 2017).

### One-Step Growth Curve

**FIGURE 4:**
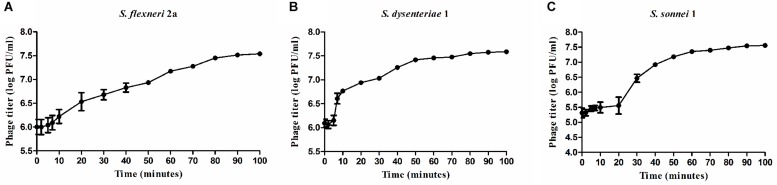
One step growth curve of bacteriophage *Sfin-1. Shigella flexneri 2a*, *Shigella dysenteriae 1*, *Shigella sonnei* were infected with *Sfin-1* at 37°C at an MOI of 0.01. After phage absorption, the cultures were diluted 10^4^-fold, incubated at 37°C and the titers in PFU per mL of *Sfin-1* from the infected cultures at different time points were determined. Result was plotted as mean ± SD (*n* = 3). **(A–C)** Present one step growth curves of *Sfin-1* in *Shigella flexneri 2a*, *Shigella dysenteriae 1*, and *Shigella sonnei*, respectively.

The one step growth curve performed at 37°C for *Sfin-1* propagated on *S. flexneri* 2a and *S. dysenteriae*1 showed a latent period of about 5 min and the average burst size was 27–28 PFU/cell (Figure 4). While in case of *S. sonnei*, the latent period was 10 min and the average burst size was estimated to be 146 PFU/cell. A phage with a large burst size can have practical advantage in therapy, because within a short period of time phage population can increase its initial dose by several 100 folds (Gallet et al., 2011).

### Whole Genome Phylogenetic and Synteny Study

**FIGURE 5:**
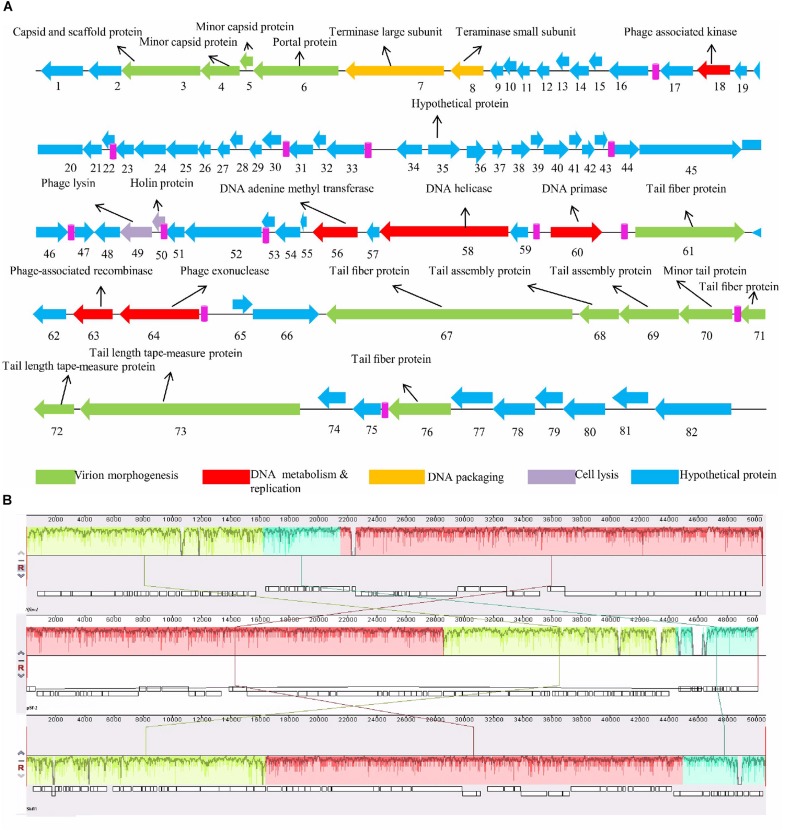
Genome organization of *Sfin-1*. **(A)** The *Sfin-1* genome map was schematically presented. The predicted CDSs are indicated as arrows, the orientation of which shows the transcription. With different colors predicted molecular function for CDS of virion morphogenesis (green arrows), DNA metabolism and replication (red arrows), DNA packaging (yellow arrows), cell lysis (violet arrows), hypothetical proteins (blue arrows), putative promoters (pink) are denoted. **(B)** Comparative genomic maps of phage *Sfin-1*, pSf-2, Shfl1 was constructed using the Mauve progressive alignments to determine conserved sequence regions. This alignment resulted into two large synteny locally collinear blocks (LCBs) with 28,894 bp (red) and 16,173 bp (green), one small LCB with 5,334 bp (sky), indicating DNA regions which are homologous among the genomes. Graphs inside the blocks show high similarity between the genomes. There are some non-identical genome regions which are denoted with white color inside the blocks. Although there seems to be genomic rearrangement, the block sequence remains the same across the genomes of all phages.

For better understanding of phage *Sfin-1* biology, its genome was sequenced. The whole genome sequencing study by Ion torrent reveals that the *Sfin-1* genome size is 50,403 bp with 45.20% GC content, close to 50.9% of the host’s chromosome (Wei et al., 2003). The Genome of *Sfin-1* shows total 82 protein CDSs after auto-annotation with GeneMarkS; 19 of which are rightward in orientation while others are leftward (Figure 5A). Among them 23 had annotated function (Table 2). No tRNA was found in the *Sfin-1* genome; this suggests that upon entry into the host, phage is completely dependent on the host tRNA for its protein synthesis.

**TABLE 2:** Features of the protein coding sequences of bacteriophage *Sfin-1* and homology to protein database.

**CDSs**	**start**	**Stop**	**Length (bp)**	**Predicted Functions**	**Best BLASTp match and Identity (%)**		**Conserved protein domain family**
CDS1	703	2	702	Hypothetical protein	Hypothetical protein ISF001_0035 [Shigella phage vB_SsoS-ISF002]	100	
CDS2	1240	764	477	Phage protein	Hypothetical protein Shfl1p34[Shigella virus Shfl1]	92	
CDS3	2364	1252	1113	Phage capsid and scaffold	Major capsid protein [Shigella phage SH6]	99	pfam09979 COG3566
CDS4	3026	2367	639	Phage minor capsid protein	Minor capsid protein [Shigella phage SH6]	99	COG2369 pfam04233TIGR01641
CDS5	3127	3011	117	Phage minor capsid protein	Minor capsid protein [Shigella phage SH6]	97	
CDS6	4400	3117	1284	Phage portal protein	Putative portal protein [Shigella virus Shfl1]	98	pfam06381 TIGR01555COG3567
CDS7	6025	4457	1569	Phage terminase, large subunit	Putative terminase large subunit [Shigella virus Shfl1]	99	COG5410 TIGR0160COG5362 pfam03237
CDS8	6589	6065	525	Phage terminase, small subunit	Putative terminase small subunit [Shigella virus Shfl1]	99	pfam16677
CDS9	6886	6674	213	Phage protein	Hypothetical protein JMPW1_023 [Escherichia phage JMPW1]	97	
CDS10	7087	6902	186	Phage protein	Hypothetical protein pSf2_045 [Shigella phage pSf-2]	98	
CDS11	7229	7068	162	Phage protein	Hypothetical protein pSf2_046 [Shigella phage pSf-2]	98	
CDS12	7597	7394	204	Phage protein	Hypothetical protein pSf2_047 [Shigella phage pSf-2]	97	
CDS13	7827	7597	231	Phage protein	Hypothetical protein JMPW1_019 [Escherichia phage JMPW1]	100	
CDS14	8171	7827	345	Phage protein	Hypothetical protein B508_00140 [Escherichia phage ADB-2]	95	
CDS15	8377	8168	210	Phage protein	Hypothetical protein JMPW2_017 [Escherichia phage JMPW2]	99	
CDS16	9022	8450	573	JK_59P	Hypothetical protein T1p62 [Escherichia virus T1]	97	
CDS17	9134	9024	111	Phage protein	Hypothetical protein [Escherichia phagevB_EcoS_SH2]	92	
CDS18	9607	9131	477	3′-phosphatase, 5′-polynucleotide kinase, phage-associated	3′-phosphatase, 5′-polynucleotide kinase [Escherichia phagevB_EcoS_SH2]	95	cd07502 PHA02530 pfam03767TIGR01675
CDS19	10193	9747	447	Phage protein	hypothetical protein SH6_0017 [Shigella phage SH6]	85	
CDS20	11283	10627	657	Phage protein	Hypothetical protein [Escherichia phagevB_EcoS_SH2]	78	
CDS21	11567	11280	288	Hypothetical protein	Hypothetical protein pSf2_056 [Shigella phage pSf-2]	97	
CDS22	11811	11551	261	hypothetical protein	Hypothetical protein B508_00105 [Escherichia phage ADB-2]	96	
CDS23	12075	11926	150	Phage protein	Hypothetical protein [Escherichia phagevB_EcoS_SH2]	96	
CDS24	12560	12075	486	Phage protein	Hypothetical protein T1p70[Escherichia virus T1]	88	
CDS25	13135	12632	504	Phage protein	Hypothetical protein B508_00095 [Escherichia phage ADB-2]	88	
CDS26	13338	13132	207	Hypothetical protein	Hypothetical protein Shfl1p10 [Shigella virus Shfl1]	99	
CDS27	13600	13409	192	Hypothetical protein	Hypothetical protein JMPW2_006 [Escherichia phage JMPW2]	96	
CDS28	13783	13610	174	Phage protein	Hypothetical protein pSf2_062 [Shigella phage pSf-2]	98	
CDS29	13890	13780	111	Phage protein	Hypothetical protein SH6_0007 [Shigella phage SH6]	100	
CDS30	14114	13887	228	Phage protein	Hypothetical protein B508_00085 [Escherichia phage ADB-2]	99	
CDS31	14351	14121	231	Phage protein	Hypothetical protein B508_00080 [Escherichia phage ADB-2]	91	
CDS32	14900		14430	Phage protein	Hypothetical protein [Escherichia phagevB_EcoS_SH2]	90	
CDS33	15097	14903	195	Phage protein	Hypothetical protein Shfl1p02 [Shigella virus Shfl1]	100	
CDS34	15703	15188	516	Phage protein	Hypothetical protein Shfl1p01 [Shigella virusShfl1]	90	
CDS35	16391	16603	213	Hypothetical protein	Hypothetical protein [Escherichia phagevB_EcoS_SH2]	100	
CDS36	16600	17304	705	Phage protein	DNA methylase [Shigella phage SH6]	97	COG0270
CDS37	17370	17705	336	Hypothetical protein	Hypothetical protein SH6_0079 [Shigella phage SH6]	93	
CDS38	17779	17940	162	Hypothetical protein	Hypothetical protein Shfl1p82 [Shigella virus Shfl1]	92	
CDS39	17970	18386	417	Phage protein	Hypothetical protein JMPW1_074 [Escherichia phage JMPW1]	93	
CDS40	18379	18579	201	Phage protein	Hypothetical protein T1p02 [Escherichia virus T1]	98	
CDS41	18596	18949	354	Phage protein	Hypothetical protein pSf2_078 [Shigella phage pSf-2]	99	
CDS42	19133	19357	225	Phage protein	Hypothetical protein [Escherichia phagevB_EcoS_SH2]	97	
CDS43	19361	19570	210	Phage protein	Hypothetical protein B508_00010 [Escherichia phage ADB-2]	100	
CDS44	19651	20067	417	Phage protein	Hypothetical protein B508_00005 [Escherichia phage ADB-2]	98	
CDS45	20144	21712	1569	Phage protein	Hypothetical protein [Escherichia phagevB_EcoS_SH2]	100	
CDS46	21717	22124	408	Phage protein	Hypothetical protein [Escherichia phagevB_EcoS_SH2]	99	
CDS47	22314	22529	216	hypothetical protein	Hypothetical protein SH6_0068 [Shigella phage SH6]	94	
CDS48	22946	22542	405	Phage protein	Hypothetical protein T1p11 [Escherichia virus T1	89	
CDS49	23431	22943	489	Phage lysin (EC 3.2.1.17)	Endolysin [Shigella phage SH6]	99	cd00737 COG3772 pfam00959
CDS50	23709	23431	279	Phage holin	Hypothetical protein T1p14 [Escherichia virus T1]	100	
CDS51	23941	23765	177	Phage protein	Hypothetical protein B508_00370 [Escherichia phage ADB2]	99	
CDS52	25156	24005	1152	Phage protein	Hypothetical protein pSf2_07 [Shigella phage pSf-2]	97	
CDS53	25540	25235	306	JK_65P	Hypothetical protein pSf2_07 [Shigella phagepSf-2]	94	
CDS54	25926	25717	210	Phage protein	Hypothetical protein B508_00360 [Escherichia phage ADB-2]	94	
CDS55	26244	25993	252	Phage protein	Hypothetical protein B508_00355 [Escherichia phage ADB-2]	99	
CDS56	26954	26241	714	DNA adenine methyltransferase, phage-associated	DNA N-6-adenine-methyltransferase [Escherichia phage ADB-2]	98	TIGR01712pfam05869
CDS57	27438	27022	417	Phage protein	VRR-NUC domain-containing protein [Escherichia phage ADB-2]	100	pfam08774 smart00990
CDS58	29453	27435	2019	DNA helicase, phage-associated	Putative ATP-dependent helicase [Shigella virus Shfl1]	99	COG 1062 cd18799 pfam 0851
CDS59	29548	30000	453	Phage protein	Hypothetical protein Shfl1p58 [Shigella virus Shfl1]	99	pfam1549
CDS60	30061	30996	936	DNA primase	DNA primase/helicase [Escherichia phage JMPW1]	99	smart00778 pfam08273 COG4643 pfam02655
CDS61	31097	32881	1785	Phage tail fiber protein	Putative tail fiber [Shigella virus Shfl1]	94	pfam 13884 PHA 00430
CDS62	33323	32910	414	Phage protein	Hypothetical protein Shfl1p55 [Shigella virus Shfl1]	98	
CDS63	34017	33370	648	Phage-associated recombinase	Putative recombination protein [Shigella phage vB_SsoS-ISF002	98	
CDS64	35156	34092	1065	Phage exonuclease	Hypothetical protein B508_00305 [Escherichia phage ADB-2]	99	pfam12684
CDS65	35683	35913	231	Hypothetical protein	Hypothetical protein pSf2_020 [Shigella phage pSf-2]	97	
CDS66	35916	36872	957	Phage protein	Hypothetical protein pSf2_021 [Shigella phage pSf-2]	99	
CDS67	40349	36900	3450	Tail fiber protein	Putative tail fiber protein [Shigella virus Shfl1]	98	COG 4733 pfam13550
CDS68	41026	40427	600	Phage tail assembly protein	Tail assembly protein [Escherichia phageJMPW1]	100	COG 4723 pfam 06805
CDS69	41757	41023	735	Phage tail assembly protein	Tail assembly protein [Escherichia phageJMPW1]	99	cd 8073 pfam 00877
CDS70	42566	41754	813	Minor tail protein	Putative minor tail protein [Escherichia phage JMPW2]	99	pfam05100 COG 4672 TIGR01600
CDS71	42968	42615	354	Tail fiber protein	Tail fiber protein [Escherichia phage JMPW1]	99	pfam 05939 COG4718
CDS72	43038	42955	84	Phage tail length tape-measure protein 1	Putative tail tape measure protein [Shigella virus Shfl1]	96	pfam06791COG5281 TIGR01541pfam09718 PRK03918
CDS73	45842	43035	2808	Phage tail length tape-measure protein 1	Putative tail tape measure protein [Shigella virus Shfl1]	98	pfam06791COG5281 TIGR01541pfam09718 PRK03918
CDS74	46152	45883	270	Phage protein	Hypothetical protein pSf2_028 [Shigella phage pSf-2]	99	
CDS75	46517	46200	318	Phage protein	Hypothetical protein ISF001_0027 [Shigella phage vB_SsoS-ISF002]	99	
CDS76	47299	46631	669	Phage tail fibers	Tail fiber protein [Escherichia phage JMPW1]	97	pfam08813
CDS77	47699	47301	399	JK_18P	Hypothetical protein pSf2_031 [Shigella phage pSf-2]	99	
CDS78	48132	47689	444	Phage protein	Hypothetical protein pSf2_032 [Shigella phage pSf-2]	97	
CDS79	48496	48125	372	JK_22P	Hypothetical protein pSf2_033 [Shigella phage pSf-2]	98	
CDS80	48906	48496	411	JK_23P	Hypothetical protein B508_00220 [Escherichia phage ADB-2]	98	
CDS81	49238	48951	288	JK_24P	Hypothetical protein ISF001_0033 [Shigella phage vB_SsoS-ISF002]	97	
CDS82	50247	49288	960	Phage protein	Hypothetical protein B508_00210 [Escherichia phage ADB-2]	98	

The whole genome Basic Local Alignment Search Tool (BLAST) analysis of *Sfin-1* against the NCBI data base showed that *Sfin-1* is related to two phages i.e., Shigella phage Shfl1 (GenBank accession number: NC_015456) and Shigella phage pSf-2 (GenBank accession number: KP085586). The phage *Sfin-1* genome sequence shares 91 and 92% nucleotide identity with Shigella phages Shfl1 and pSf-2, respectively. Genomic features of three phages ware compared in Supplementary Table S2. Though the genome sizes, GC contents, number of transcription terminator sequences and CDSs are quite similar and genes of predicted structural and functional proteins share high degree of homology, they are differently arranged and their orientations are sometimes opposite (Supplementary Figure S1). Maximum differences present into their hypothetical proteins which are uncharacterized according to database. Around 75% genes of *Sfin-1* are of unknown functions while most of them have >80% homology with their counterparts in Shfl1 and Psf-2 genome. Since these phages were isolated from different geographical locations, the high degree of similarity probably appears from their complex evolutionary relationships with their common host *S. flexineri* (Shen et al., 2016). Since the genome of *Sfin-1* diverges from the other phage genomes, with similarity searches only a fraction (28.04%) of protein functions could be predicted emphasizing the novelty of this phage. Therefore, a thorough investigation is needed to fully understand its biology. The mauve alignment of *Sfin-1*, Shfl1 and pSf-2 resulted into two large synteny locally collinear blocks (LCBs) with 28,894 bp (red) and 16,173 bp (green), one small LCB with 5,334 bp (sky), indicating DNA regions which are homologous among the genomes. Graphs inside the blocks show high similarity between the genomes. There are some non-identical genome regions which are denoted with white color inside the blocks. Although there seems to be genomic rearrangement, the block sequence remains the same across the genomes of all phages.

Moreover the alignment of the three phages also demonstrated that some regions are highly homologous with significant rearrangements (Figure 5B). This indicated that these phages share a common genome organization although positions of the genes are different.

### Module Analysis

The annotated proteins of *Sfin-1* can be categorized into the following functional groups: DNA metabolism and replication proteins; this module presents in the middle part of the *Sfin-1* genome. They are 3′-phosphatase, 5′-polynucleotide kinase/CDS18, Phage associated N-6-DNA adenine-methyl transferase/CDS56, DNA helicase/CDS58, DNA primase/helicase/CDS60, and phage associated recombinase/CDS63, phage exonuclease/CDS64). The 3′-phosphatase, 5′-polynucleotide kinase belongs to the family that includes the C-terminal domain of the bifunctional enzyme T4 polynecleotide kinase/phosphatase PNKP. The PNKP phosphatase domain can catalyze the hydrolytic elimination of the 3′-phosphoryl group of DNA, RNA and deoxynucleoside 3′-monophosphates. The enzyme N-6-DNA adenine-methyl transferase (DAM) is involved in methylation of GATC sequence of its own DNA to protect it from exonuclease. The counterpart of this enzyme of *Sfin-1* is present in Escherichia phage ADB-2 (99% identity). There are two helicase coding genes in the *Sfin-1* phage genome; one is ATP dependent and has 99% identity with Shigella phage Shfl1 helicase and another is primase associated having 99% identity with Escherichia phage JMPW1. The primase/helicase protein has a zinc finger motif at its N terminal region and ATP binding region at its C-terminal part with origin recognition property. CDS63 contains a protein which encodes phage associated recombinase domain that is commonly found associated with Pfam04404 of ERF superfamily. The family includes single strand annealing proteins (SSAPs), such as Rad52, ERF, Red-beta, and RecT that function in RecA independent and RecA dependent DNA recombination pathways. This type of phage encoded recombinase are mainly involved in horizontal gene transfer by homologous recombination, thus promotes gene shuffling among phages which accelerates evolution. The phage exonuclease acts together with recombinase and involves into replication process from fork structures as well as in nucleotide metabolism. CDS64 encodes an exonuclease VIII that is related to PDDEXK superfamily. Thus 3′-phosphatase, 5′-polynucleotide kinase, phage recombinase, exonuclease are involved in DNA metabolism and recombination process of the phage genome after entering into host cells.

Sequenced based prediction of the phage *Sfin-1* genome identified that upstream and downstream cluster genes are involve in viral head morphogenesis and tail component formation, respectively. CDS3, CDS4, and CDS5 which are likely to produce phage capsid and scaffold protein belong to Phage Mu F like protein family. Members of this family are required for viral head morphogenesis. CDS6 encodes head and tail junction portal protein that is believed to form the pore through which genome is packaged into the prohead and is also a part of the packaging motor (Lokareddy et al., 2017). CDS7, CDS8 encode phage large and small subunit, respectively, which are involved in packaging of the concatameric DNA in phage capsids (Mobberley et al., 2008). Apart from upstream genes, the downstream genes CDS61, CDS67, CDS70, CDS71, CDS72, CDS73, and CDS 76 probably encode the tail component whereas CDS68 and CDS69 direct the synthesis of protein responsible for tail assembly. CDS72 and CDS73 that encodes tail tape measure protein is the second largest gene of the phage genome. Although genomes of phages *Sfin-1*, Shfl1 and pSf-2 were found to be highly similar as discussed above, they infect different species (Supplementary Table S2). As tail fiber proteins are involved in host range determination (Yang et al., 2018), the sequence and predicted features of three phages’ tail fiber proteins were compared. Four tail fiber proteins of *Sfin-1* are (A) CDS61, (B) CDS67, (C) CDS71, and (D) CDS76. Their counterparts in Shfl1 are CDS53 (93.60% identity), CDS47 (98% identity), CDS43 (95.73% identity), CDS39 (96.85% identity), respectively and in pSf-2 are CDS16 (97.31% identity), CDS22 (98% identity), CDS26 (94.02% identity), CDS30 (100% identity), respectively. The tail fiber proteins of these phages not only share high degree of nucleotide sequence homology, the positions of helix and strands in them are also very similar.

However, 69, 116, 239, 277, 313, 345, 380, 415, 483, and 545 of tail fiber protein A, 43, 48, 72, 101, 152, 158, 190, 198, 199, and 1,089 for tail fiber protein B, 8, 61, and 67 for tail fiber protein C amino acid residues of *Sfin-1* sequence differ to those in phages Shfl1 and Psf-2, which might result in differences in their protein binding regions (Supplementary Figures S2–S5) and ultimately determine the changes in host specificity. No difference of amino acid residue of tail fiber protein D sequence has been noticed.

Tail length of the lambdoid phages corresponds to the length of the tail tape measure protein where single amino acid is equivalent to ∼0.15 nm (Katsura, 1990). According to this hypothesis the tail length of the phage *Sfin-1* is approximately about 144 nm long which is approximately close to our measured length 155 nm. Most of the virion morphogenesis genes encode proteins which show similarity to either Shigella phage psf-2 or Shigella virus Shfl1 encoded proteins. However, gene order of Shfl1 is totally reverse to the *Sfin-1* while psf-2 possesses the same gene orientation but at different positions. The terminase subunits and CDS7-CDS8, the DNA packaging genes of *Sfin-1* have counterparts in Shigella phage Shfl1 with 99% identity. These proteins are mainly involved in ATP dependent DNA packaging system.

**FIGURE 6:**
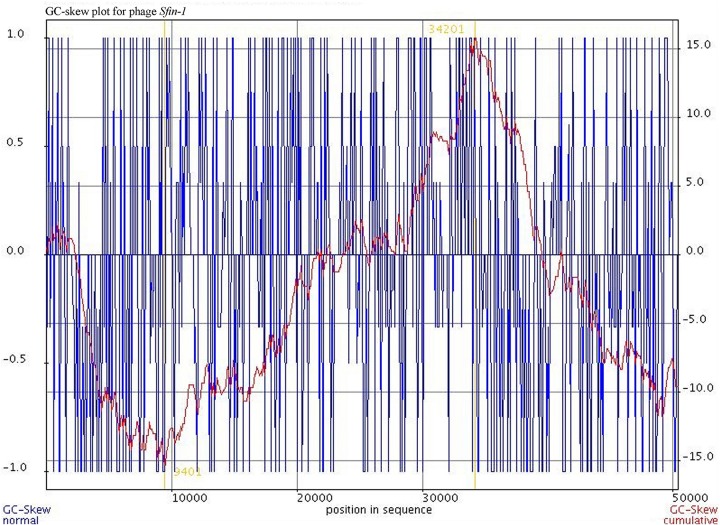
Cumulative GC skew analysis of *Sfin-1* genome sequence. The cumulative graph displays the global minimum and maximum. The window size of 1,000 bp and a step size of 100 bp were used to calculate the global minimum and maximum. The blue and red lines represent the GC-skew and the cumulative GC-skew, respectively. The putative origin of replication (9,401 nt) and the putative terminus location (34,201 nt) can be predicted from the minimum and maximum of a GC-skew.

Cell lysis proteins, phage lysine/CDS49 and holin/CDS50 are present in *Sfin-1* phage genome. These genes are crucial for host cell destruction during burst step of phage life cycle. Once the new phage progeny has been assembled, most of the phages lyse their host by using a dual lysis system, which contains a pore forming protein holin and cell wall degrading enzyme phage lysozyme or endolysin. CDS49 and CDS50 located contagiously at the middle part of the *Sfin-1* genome that are involved in cell lysis. CDS49 encodes 162 amino acids long phage lysozyme/endolysin belonging to the pfam00959 family found in dsDNA phages. Members of this family in conjunction with holin (CDS50) cleave the ß1,4-glycosidiclinkage of polysaccharide present in the bacterial membrane (Ziedaite et al., 2005). BLASTp analysis also indicates the presence of one DNA binding transcriptional regulatory cro protein encoded by CDS59 belonging to the HTH_XRE superfamily. *Sfin-1* may use this protein to regulate transcriptional timing in the gene expression. So presence of lysis genes but no lysogeny related genes into the *Sfin-1* genome clearly indicates that this phage is a potent lytic phage. The GeneSkew program, an application for computing and plotting nucleotide skew data predicted the probable replication origin of *Sfin-1*. The GC-skew plot (Figure 6) indicates that the replication origin could be the region around the nucleotide 34201 (close to CDS64).

### Proteomic Analysis

The purified phage of *Sfin-1* was analyzed by liquid chromatography/tandem mass spectrometry (LC-MS/MS). The LC-MS/MS analysis detected 22 phage proteins including 16 structural with a coverage ranging from 10 to 25% and 6 functional with a coverage ranging from 16 to 23% (Table 3). Among 16 structural proteins, the phage capsid and scaffold protein, minor capsid protein, tail fiber protein, phage lysin, phage holin, minor tail protein, tail assembly protein, terminase large and small subunit are associated with virion morphogenesis and phage packaging functions while the other 6 functional proteins are phage helicase, exonuclease, recombinase, 5′ polynucleotide kinase, DNA adenine methyltransferase, primase. These proteins are involved in DNA metabolism and replication functions of the phage genome after entering into host cells. The phage encoded recombinase is mainly involved in horizontal gene transfer by homologous recombination process which helps in gene rearrangement among phages resulting in acceleration of evolution.

**TABLE 3:** Virion proteins detected by LC-MS/MS.

**CDS**	**Start**	**End**	**Mass (Da)**	**Predicted function**	**BLASTp (best match)^1^**	**Identity (%)^2^**	**LC-MS/MS^3^**
03	2364	1252	40117	Phage capsid and scaffold	WP_022638357/*Lactobacillus plantarum*	10/24 (42)	25
04	3026	2367	24234	Minor capsid	WP_002314920/*Enterococcus faecium*	23/89 (26)	15
05	3127	3011	432991	Phage minor capsid	WP_024636649/*Enterococcus faecium*	10/31 (32)	13
06	4400	3117	48011	Portal protein	WP_009841304/*Vibrio* spp. AND4	15/38 (39)	25
07	6025	4457	60287	Terminase large subunit	YP_006119910/*Escherichia coli O83*	28/115 (24)	14
08	6589	6065	19320	Terminase small subunit	YP_006780198/*Escherichia coli O104*	11/24 (46)	13
18	9607	9131	17985	3′-phosphatase, 5′-polynucleotide kinase	NP_268935/*Streptococcus phage 370.1*	9/12 (75)	16
49	23431	22943	18276	Phage lysin	YP_535193/*Lactobacillus salivarius UCC118*	5/15 (33)	19
50	23709	23431	7577	Phage holin	WP_061631936/*Streptococcus pneumonia*	6/15 (40)	12
56	26954	26241	27014	DNA adenine methyltransferase	NP_268935/*Streptococcus phage 370.1*	7/22 (32)	16
58	29453	27435	76000	DNA helicase	WP_058540300/*Pseudomonas putida*	29/98 (30)	14
60	30061	30996	34612	DNA primase	WP_023241409/*Salmonella enterica*	27/76 (36)	23
61	31097	32881	64478	Tail fiber protein	NP_857641/*Yersinia pestis*	13/40 (33)	10
63	34017	33370	23935	Phage associated recombinase	WP_060441855/*Serratia marcescens*	32/126 (25)	16
64	35156	34092	40165	Phage exonuclease	YP_004243626/*Bacillus subtilis*	16/50 (32)	18
67	40349	36900	127462	Tail fiber protein	WP_010939430/*Desulfovibrio vulgaris*	109/484 (30)	21
68	41026	40427	20880	Tail assembly protein	WP_023658790/*Pseudomonas fluorescens*	23/73 (32)	20
69	41757	41023	28345	Tail assembly protein	WP_005987870/*Desulfovibrio africanus*	17/70 (24)	17
70	42566	41754	29021	Minor Tail protein	WP_007364011/*Opitutaceae bacterium TAV1*	7/23 (30)	18
71	42968	42615	13041	Tail fiber protein	WP_010939430/*Desulfovibrio vulgaris*	5/11 (45)	21
73	45842	43035	101408	Tail-length tape measure protein	WP_063259367/*Enterobacter cloacae*	63/284 (25)	22
76	47299	46631	24077	Tail fiber protein	NP_857641/*Yersinia pestis*	9/27 (33)	10

*^1^The matched homologs of LC-MS/MS data. ^2^The percentage identity is calculated based on the number of identical amino acid residues (numerator) over the number of compared residues (denominator) and shown in parentheses. ^3^The sequence coverage (%) determined by mass spectrometry of the protein is indicated.*

### Determination of the Bacteriophage Genome Ends

**FIGURE 7:**
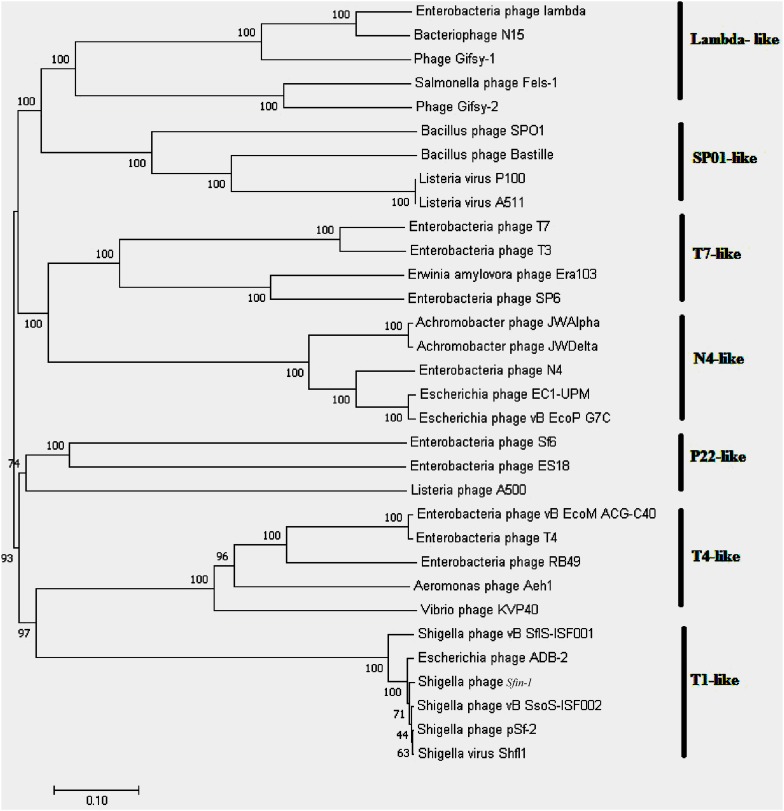
Phylogenetic tree of terminase large subunit. Phages with known packaging mechanisms were only included. Bootstrap analysis was performed with 1,000 repetitions. The terminase large subunits were compared in the MEGA 7.0 version using neighbor-joining method.

**FIGURE 8:**
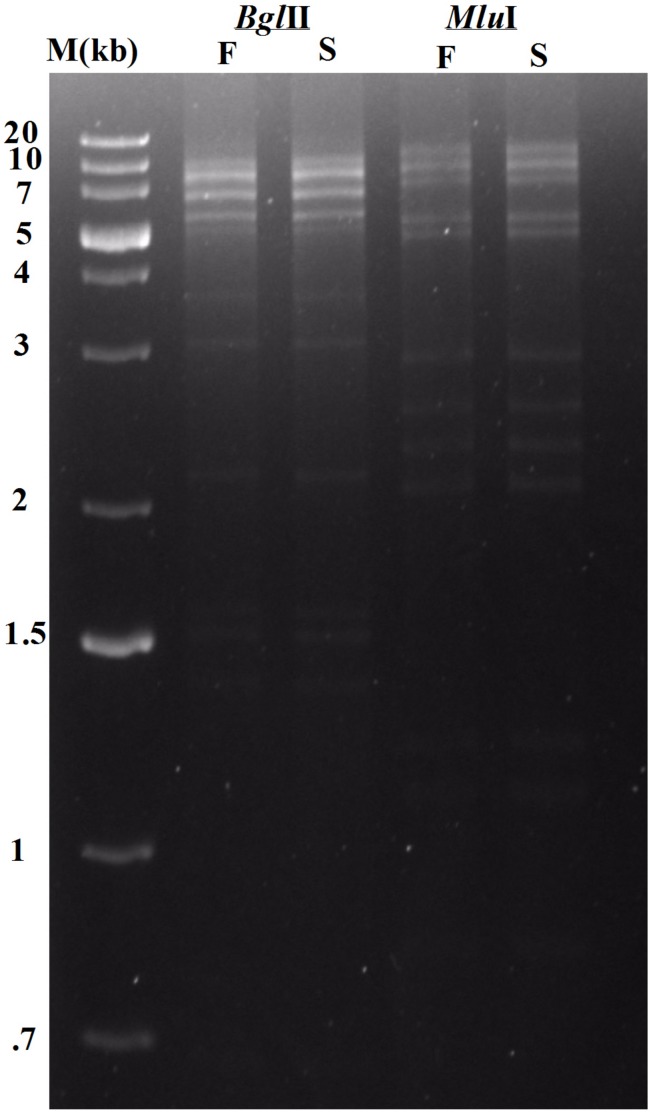
Enzymatic analysis of *Sfin-1* genomic DNA. Phage DNA was completely digested with *Bgl*II and *Mlu*I and the products were analyzed by 0.8% agarose gel electrophoresis, Lane M indicates the 1 kb Plus DNA Ladder. F and S indicate that the digests were heated to 80°C for 15 min and then cooled fast on ice or slow at room temperature, respectively.

Whole genome sequencing followed by assembly of *Sfin-1* genome initially generated a linear 50,530 bp fragment with a 127 bp terminal repeat at both of its ends. The linear genome is expected as in tailed bacteriophages within the channel of portal protein only one dsDNA can pass and therefore, the head contains linear genome. However, linear phage genome may have different types of ends. It is known that phage terminase enzyme creates the virion DNA ends and this enzyme is one of the most conserved phage proteins within the group. Therefore, comparative analysis of terminase amino acid sequence of a phage clusters it with others that generate similar ends. According to the phylogenetic analysis of the large terminase subunit, *Sfin-1* was clustered with the terminase of *E. coli* phage ADB-2, Shigella phage Shfl1 and psf-2 which belong to T1 family of phage (Figure 7). This family has double stranded terminal repeats in their chromosome ends. Based on its close relationship with the T1 like phages, it is predicted that *Sfin-1* genome is possibly circularly permutated with direct terminal repeats. For a circularly permuted headful packaging phage chromosome, the site of initiation cleavage is not precise. So, alternative initiation cuts are spread over regions on the concatemers. As a result, chromosome lengths of individual virions are imprecise. The undigested phage DNA as well as restriction pattern of these type of phages are expected to contain all the fragments from a circular genome along with a submolar pac fragment as happened with P22 genome (Casjens and Gilcrease, 2009) and in case of imprecise series initiation cleavage like phage sf6 and ES18, the pac fragment may not be detected. Instead a blur background will be observed because of the variable lengths of terminal fragments (Casjens and Gilcrease, 2009). When *Sfin-1* genomic DNA was digested with restriction endonucleases *Bgl*II and *Mlu*I, the result was in agreement with the predicted result based on a circular *Sfin-1* genome (Figure 8). Restriction digests were warmed at 80°C and then cooled down slowly or rapidly. After slow cooling single stranded cohesive ends of the phage genome are expected to anneal and appear as a longer fragment in gel electrophoresis. But no difference was noticed between slow and fast cooled sets for both the enzymes indicating absence of cohesive ends in the *Sfin-1* genome. Additionally blur background was observed in electrophoresis gel. This result indicates that *Sfin-1* is a T1 like headful packaging phage.

### Identification of Host Receptor

**FIGURE 9:**
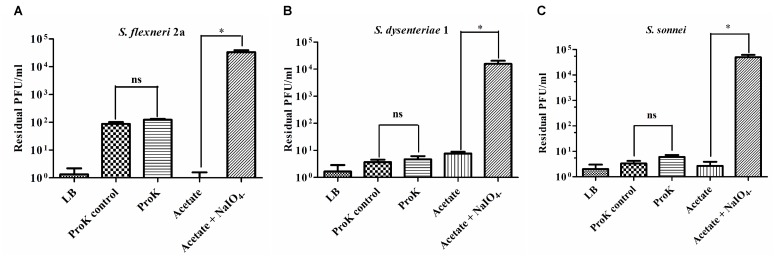
*Sfin-1* infections on proteinase K and periodate treated host. The effect of proteinase K and sodium periodate on adsorption of phage *Sfin-1*. *Shigella flexneri* 2a, *Shigella dysenteriae* 1, and *Shigella sonnei* cultures (OD600 = 0.3) were treated with proteinase K (250 mg/mL) or sodium periodate (200 mM NaIO_4_) followed by *Sfin-1* (MOI 0.0001) infection. Upon centrifugation, the phage titer in supernatant was determined as described in “Materials and Methods” section. Cells suspended in LB, cells incubated at 55°C in LB and cells in acetate buffer were used as control. The results are shown as residual PFU percentages. The phage titer in the control supernatant was set to 100%. Mean ± SD of three independent experiments are indicated. To determine the significance of the differences between group means, unpaired *t*-tests were performed between the controls and the tests. ^∗^Significance level, i.e., *P* < 0.05, “ns” indicates non-significant. **(A–C)** Results of *S. flexneri* 2a, S. *dysentariae*1, and *S. sonnei*, respectively.

The crucial step in the phage infection is its adsorption to the surface of the host through receptor. As the *Shigella* spp. belongs to the class of gram negative bacteria with complex lipopolysaccharide (LPS) and protein at its cell surface, the outer membrane carbohydrate (LPS) or protein may act as the specific receptor for phage infection. Therefore, it is very important to find out whether LPS or protein is the recognition site of phage *Sfin-1* during infection. To determine the actual receptor of the phage *Sfin-1*, outer membrane LPS and proteins of *S. flexneri* 2a, *S. dysenteriae* 1, and *S. sonnei* were degraded by periodate and proteinase K respectively prior to the infection. *Sfin-1* showed no change in infection efficiency with or without proteinase K treatment to the above mentioned hosts. In contrast, high number of phage particles remained unabsorbed when hosts were pre-treated with periodates (Figure 9). So, this experiment suggests that the adsorption of phage *Sfin-1* to the host is mediated by the outer membrane complex LPS structure. When LPS is used as phage receptor, phage shows strain specificity. Here, we see *Sfin-1*, which uses LPS as receptor to infect three *Shigella* spp. This apparent anomaly may be attributed to the close relationship between *Shigella* spp. Although there is heterogeneity among LPS’s O-antigens of *Shigella* spp. they share extensive similarity (Liu et al., 2008).

## Concluding Remarks

Multidrug-resistant *Shigella* infection has already become a critical problem in many countries. Against the backdrop of widespread antibiotic resistance, phages are re-emerging as promising therapeutic agent to control bacterial infections. In the current study we have characterized a novel thermostable and wide range pH tolerant *Siphoviridae* phage *Sfin-1* that infects and lyses the important antibiotic resistant enteropathogens *Shigella* spp. This is the first reported phage which infects both *S. flexneri* and *S. dysenteriae.* The article presents complete physical characterizations, sequence analysis and detailed genome annotation of phage *Sfin-1*. Phage structural proteins have also been identified through LC-MS/MS study. The Genomic data are important resources to study and use phages to control specific bacteria species. Phylogenetic analysis concludes that *Sfin-1* belongs to the T1-like bacteriophage and thus it may be packaged by headful packaging method. Further analysis of phage *Sfin-1* cell wall receptor revealed that, bacteriophage *Sfin-1* recognizes LPS O-antigen as its primary receptor for adsorption. Further studies on *Sfin-1* phage will be useful to apply it for therapeutic purpose against multidrug-resistant shigellosis.

## Data Availability

The datasets generated for this study can be found in the GenBank under accession number MF468274.

## Author Contributions

SA and NG conceived and designed the whole study. SD supplied the clinical samples. SA and BR performed the experiments. AG analyzed the phage structure. SA, UB, and NG analyzed the results. BB performed the statistical analysis. SA, UB, SD, BB, and NG prepared the manuscript. All authors wrote, read and approved the final manuscript.

## Conflict of Interest Statement

The authors declare that the research was conducted in the absence of any commercial or financial relationships that could be construed as a potential conflict of interest.

## Footnotes

1 http://exon.gatech.edu/GeneMark/genemarks.cgi

2 http://www.ncbi.nlm.nih.gov/

3 http://genskew.csb.univie.ac.at/

4 https://www.fruitfly.org/seq_tools/promoter.html

5 http://lowelab.ucsc.edu/tRNAscan-SE/

6 http://asap.ahabs.wisc.edu/mauve/

## Supplementary Material

FIGURE S1 Comparative genomic analysis of *sfin-1* with Shfl1 and pSf-2. Comparison of *Sfin-1* genome with that of **(A)** Shfl1 and **(B)** pSf-2 are schematically presented. Green (virion morphogenesis), yellow (DNA packaging), red (DNA metabolism and replication), and gray (cell lysis) colors represent different groups of homologous proteins of known functions. Numbers within parentheses indicate degree of homology as calculated via tblastx. The violet arrow heads indicate *Sfin-1*-specific genes which are absent in shfl1 and pSf-2. Black arrowheads denote genes of Shfl1 and pSf-2 absent in *Sfin-1*. Hypothetical proteins having more than 90% homology are indicated by saffron color and less than 90% by sky blue color.

FIGURE S2 The comparison of tail fiber protein sequence A (CDS61) of *Sfin-1*, CDS53 of Shfl1 and CDS16 of pSf-2. **(A)** The predicted features comparison of three proteins. The red diamond represents protein binding region, the red and blue rectangles represent the helix and strand of proteins, respectively. **(B)** The comparison of amino acid sequences of three proteins by ClustalW. ^∗^Represents same amino acid and blank represents different amino acids.

FIGURE S3 The comparison of tail fiber protein sequence B (CDS67) of *Sfin-1*, CDS47 of Shfl1 and CDS22 of pSf-2. **(A)** The predicted features comparison of three proteins. The red diamond represents protein binding region, the red and blue rectangles represent the helix and strand of proteins, respectively. **(B)** The comparison of amino acid sequences of three proteins by ClustalW. ^∗^Represents same amino acid and blank represents different amino acids.

FIGURE S4 The comparison of tail fiber protein sequence C (CDS71) of *Sfin-1*, CDS43 of Shfl1 and CDS26 of pSf-2. **(A)** The predicted features comparison of three proteins. The red diamond represents protein binding region, the red and blue rectangles represent the helix and strand of proteins, respectively. **(B)** The comparison of amino acid sequences of three proteins by ClustalW. ^∗^Represents same amino acid and blank represents different amino acids.

FIGURE S5 The comparison of tail fiber protein sequence D (CDS76) of *Sfin-1*, CDS39 of Shfl1 and CDS30 of pSf-2. **(A)** The predicted features comparison of three proteins. The red diamond represents protein binding region, the red and blue rectangles represent the helix and strand of protein, respectively. **(B)** The comparison of amino acid sequences of three proteins by ClustalW. ^∗^Represents same amino acid and blank represents different amino acids.

TABLE S1 Comparative analysis of *Sfin-1* with different other Shigella phages available in NCBI database. NA^∗^ = no data available.

TABLE S2 Comparative analysis of *Sfin-1* with pSf-2 and Shfl1 genomes.
