# Supplementary figures and images for: Genomic and Proteomic Characterizations of Sfin-1, a Novel Lytic Phage Infecting Multidrug-Resistant Shigella spp. and Escherichia coli C
Source: Front Microbiol. 2019 Aug 22;10:1876. doi: 10.3389/fmicb.2019.01876 (PMC6714547; doi:10.3389/fmicb.2019.01876)

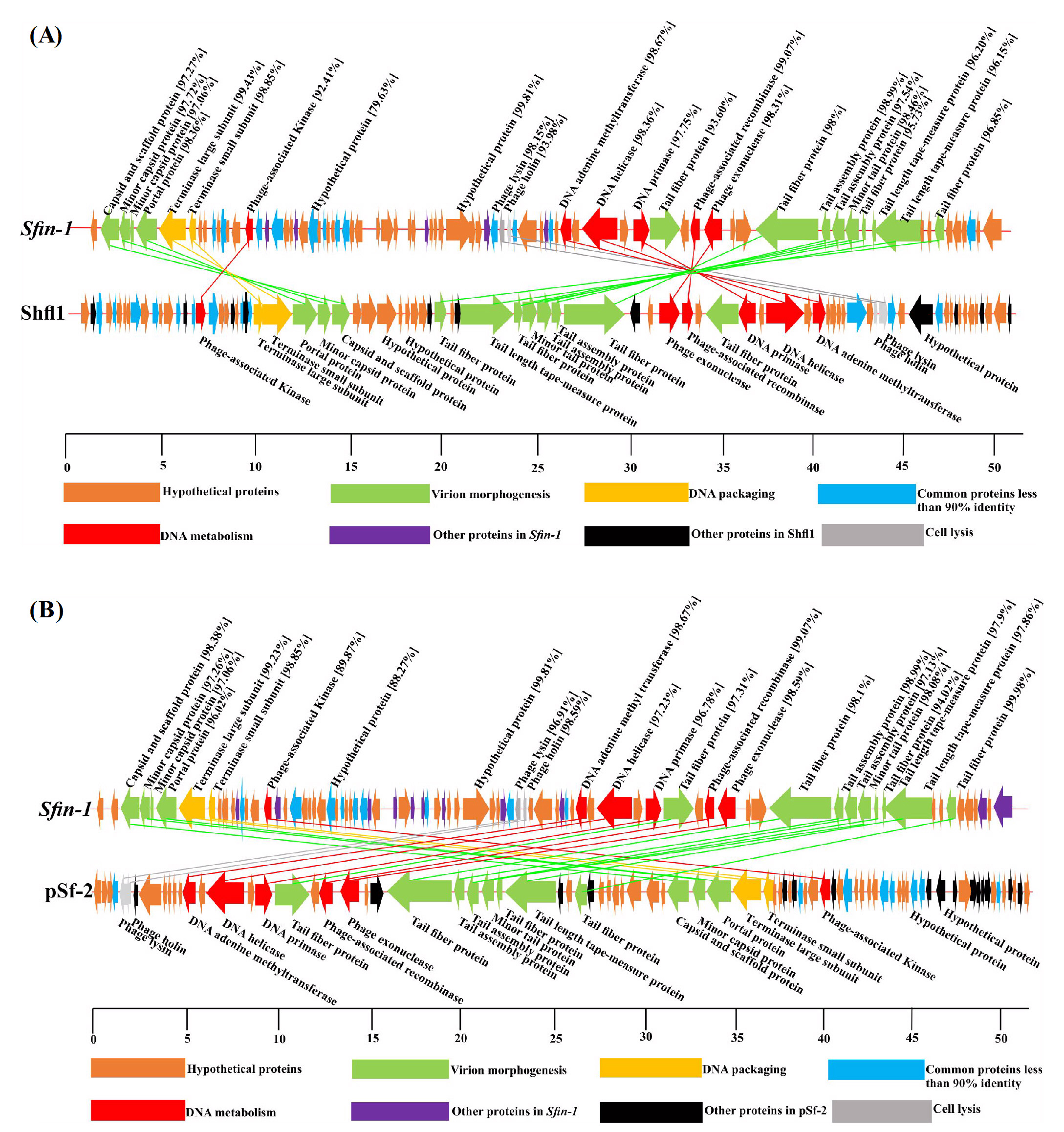

Supplement: FIGURE S1 — Comparative genomic analysis of sfin-1 with Shfl1 and pSf-2. Comparison of Sfin-1 genome with that of (A) Shfl1 and (B) pSf-2 are schematically presented. Green (virion morphogenesis), yellow (DNA packaging), red (DNA metabolism and replication), and gray (cell lysis) colors represent different groups of homologous proteins of known functions. Numbers within parentheses indicate degree of homology as calculated via tblastx. The violet arrow heads indicate Sfin-1-specific genes which are absent in shfl1 and pSf-2. Black arrowheads denote genes of Shfl1 and pSf-2 absent in Sfin-1. Hypothetical proteins having more than 90% homology are indicated by saffron color and less than 90% by sky blue color. [file Image_1.JPEG]

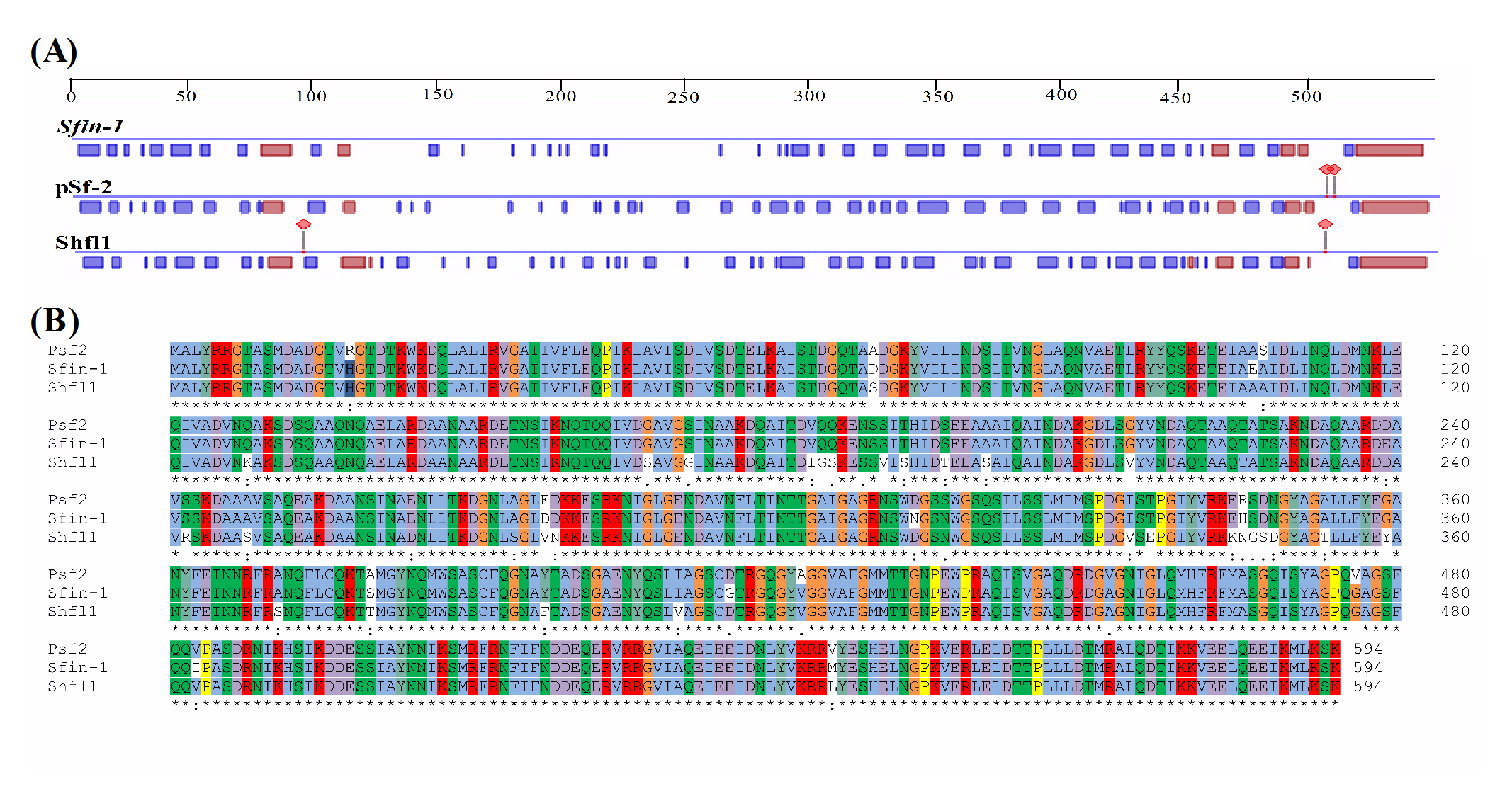

Supplement: FIGURE S2 — The comparison of tail fiber protein sequence A (CDS61) of Sfin-1, CDS53 of Shfl1 and CDS16 of pSf-2. (A) The predicted features comparison of three proteins. The red diamond represents protein binding region, the red and blue rectangles represent the helix and strand of proteins, respectively. (B) The comparison of amino acid sequences of three proteins by ClustalW. ∗Represents same amino acid and blank represents different amino acids. [file Image_2.JPEG]

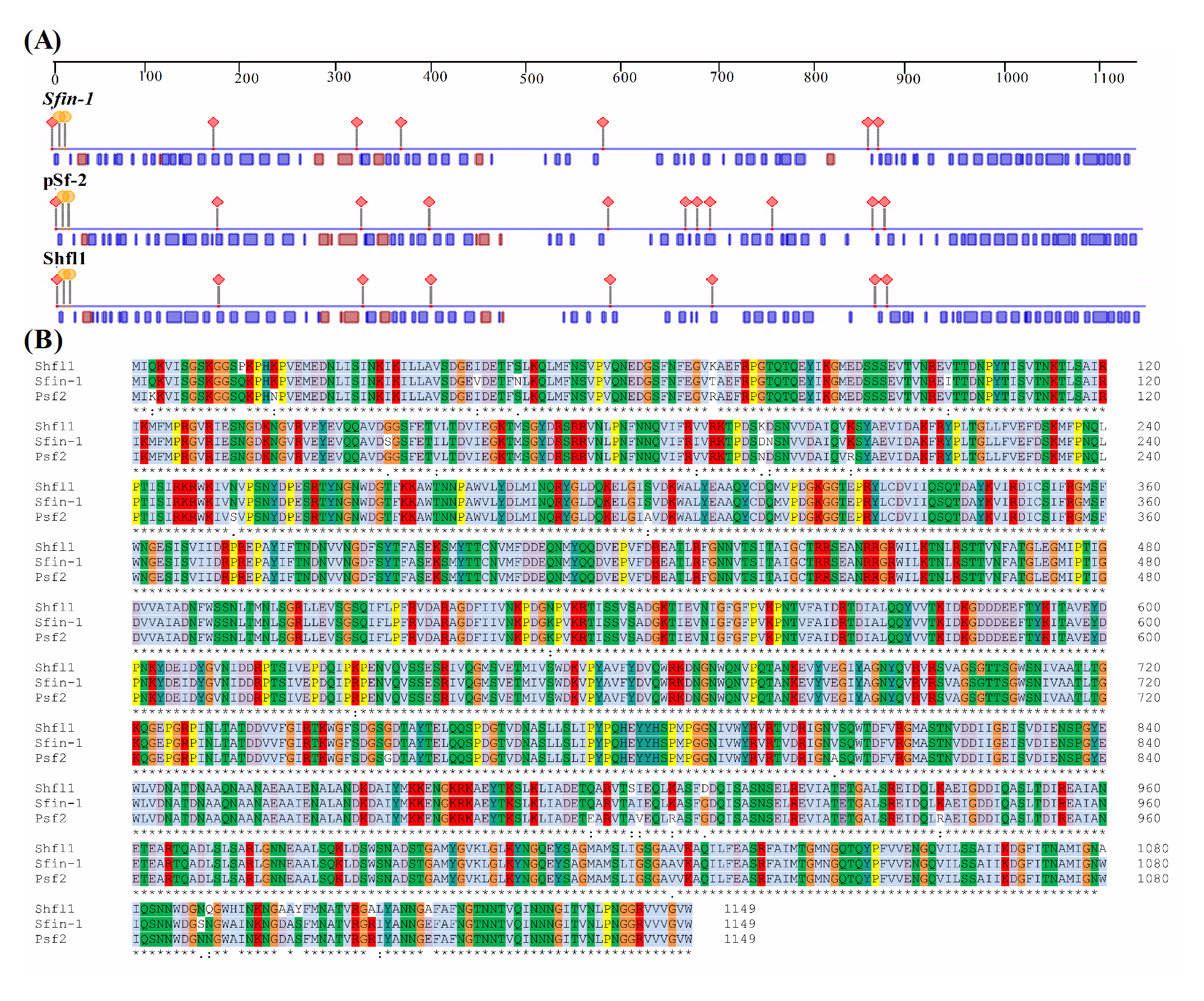

Supplement: FIGURE S3 — The comparison of tail fiber protein sequence B (CDS67) of Sfin-1, CDS47 of Shfl1 and CDS22 of pSf-2. (A) The predicted features comparison of three proteins. The red diamond represents protein binding region, the red and blue rectangles represent the helix and strand of proteins, respectively. (B) The comparison of amino acid sequences of three proteins by ClustalW. ∗Represents same amino acid and blank represents different amino acids. [file Image_3.JPEG]

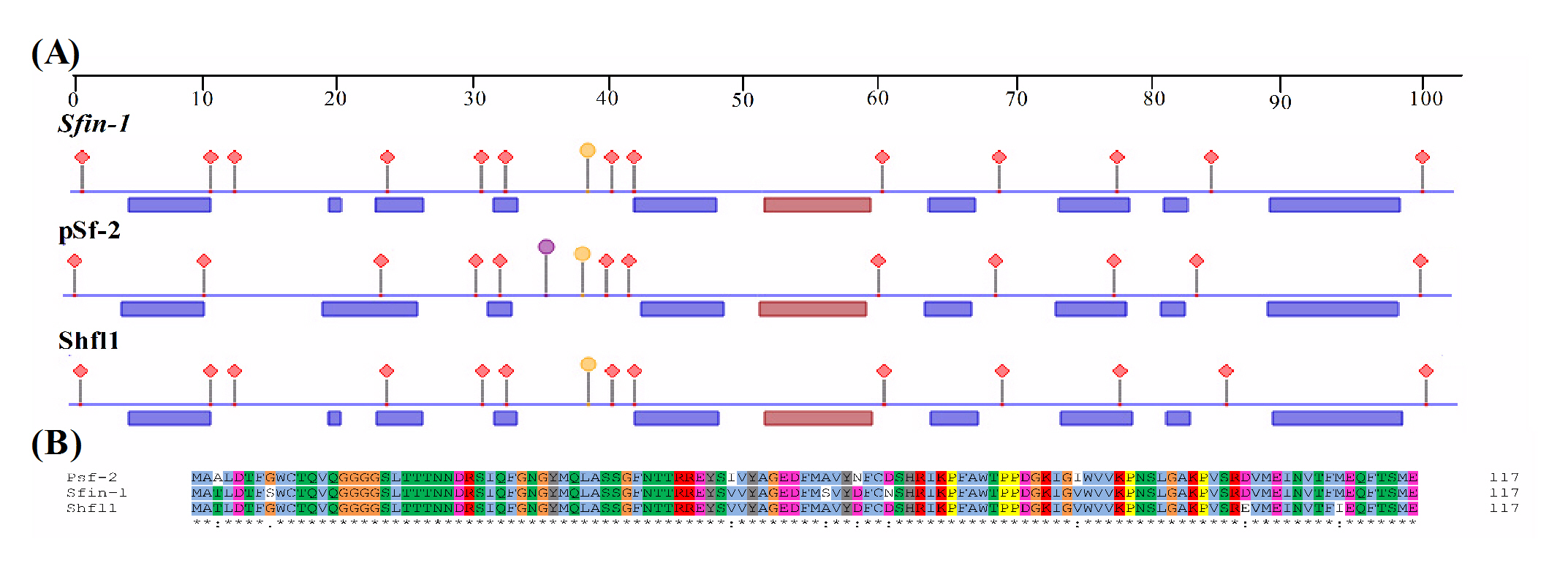

Supplement: FIGURE S4 — The comparison of tail fiber protein sequence C (CDS71) of Sfin-1, CDS43 of Shfl1 and CDS26 of pSf-2. (A) The predicted features comparison of three proteins. The red diamond represents protein binding region, the red and blue rectangles represent the helix and strand of proteins, respectively. (B) The comparison of amino acid sequences of three proteins by ClustalW. ∗Represents same amino acid and blank represents different amino acids. [file Image_4.JPEG]

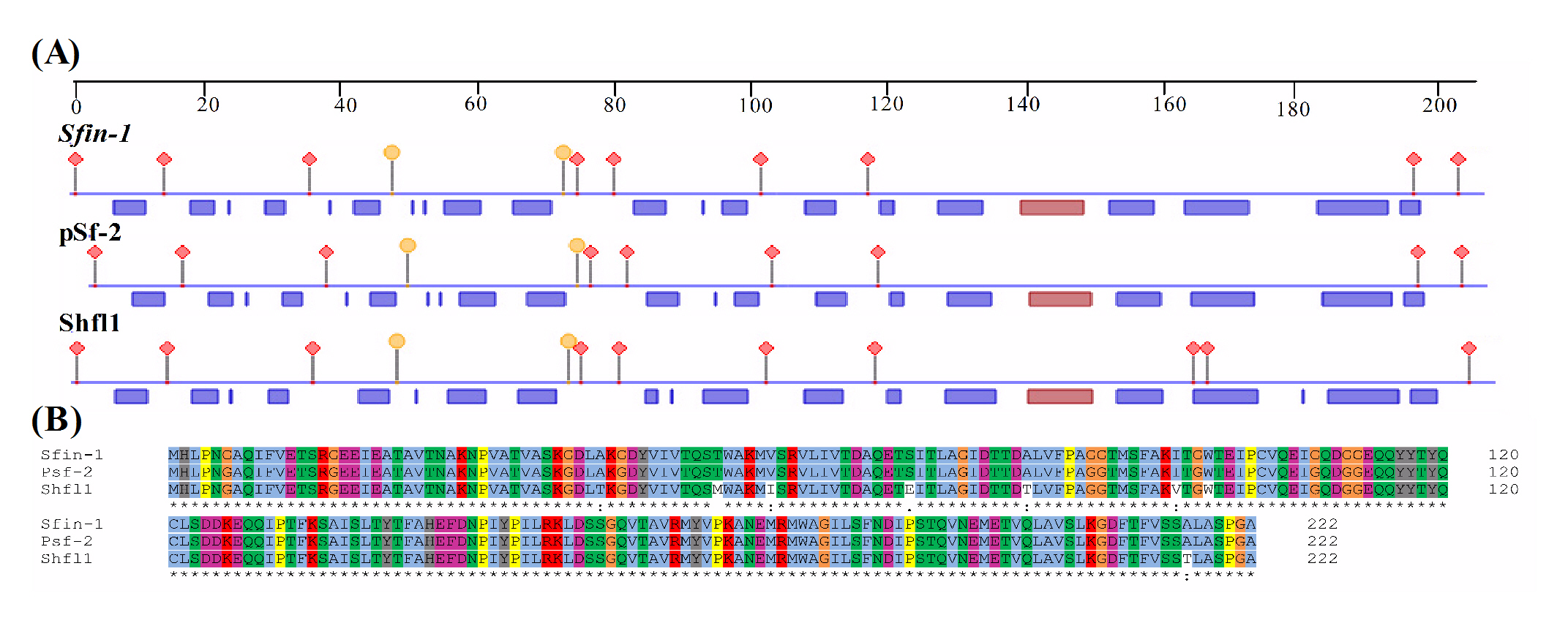

Supplement: FIGURE S5 — The comparison of tail fiber protein sequence D (CDS76) of Sfin-1, CDS39 of Shfl1 and CDS30 of pSf-2. (A) The predicted features comparison of three proteins. The red diamond represents protein binding region, the red and blue rectangles represent the helix and strand of protein, respectively. (B) The comparison of amino acid sequences of three proteins by ClustalW. ∗Represents same amino acid and blank represents different amino acids. [file Image_5.JPEG]
