# Supplementary material for: Genomic and Proteomic Characterizations of Sfin-1, a Novel Lytic Phage Infecting Multidrug-Resistant Shigella spp. and Escherichia coli C
Source: Front Microbiol. 2019 Aug 22;10:1876. doi: 10.3389/fmicb.2019.01876 (PMC6714547; doi:10.3389/fmicb.2019.01876)
Supplement: TABLE S1 — Comparative analysis of Sfin-1 with different other Shigella phages available in NCBI database. NA∗ = no data available. [file Table_1.doc]

**Supplementary TABLE S1**

| **Phage Name** | **GeneBank accession no.** | **Genome length**  **(bp)** | **Genome type** | **Host range** | **MOI** | **Burt size (PFU/cell)** | **Reference** |
| --- | --- | --- | --- | --- | --- | --- | --- |
| **Shigella phage *Sfin-1*** | **MF468274** | **50403 bp** | **Linear** | ***Shigela flexneri,***  ***Shigella dyscenteriae,***  ***Shigella sonnei* and**  ***E. coli C*** | **0.01** | **28-146** | **This study** |
| Shigella phage SFPH2 | MH464253 | 40387 bp | Linear | *Shigella flexneri* 2a | 0.1 | 30 |  |
| Shigella phage SH6 | KX828710 | 50552 bp | Linear | *Shigella flexneri,*  8 strains of *E.coli* | 0.05 | 103± 16 |  |
| Shigella phage SH7 | KX828711 | 164870 bp | Linear | *Shigella flexneri,*  *E.coli* O157:H7,  *Shigella dysenteriae, Salmonella paratyphi*. | 0.05 | 26±5 |  |
| Shigella phage pSb-1 | KF620435 | 71629 bp | Circular | *Shigella boydii* | 0.01 | 152.6 |  |
| Shigella phage pSf-2 | KP085586 | 50109 bp | Circular | *Shigella flexneri* | 0.01 | 16 |  |
| Shigella phage vB_SdyS-ISF003 | MH719028 | 62000 bp | Linear | *Shigella dysenteriae* | 0.01 | 128 ± 12 |  |
| Shigella phage vB_SflS-ISF001 | MG049919 | 50552 bp | Linear | *Shigella flexneri* | 0.1 | 53 ± 4 |  |
| Shigella phage vB_SsoS-ISF002 | MF093736 | 50564 bp | Linear | *Shigella flexneri,*  *Shigella sonnei* | 0.1 | 76±9 |  |
| Shigella phage pSf-1 | KC710998 | 51821 bp | Linear | *Shigella flexneri* | 0.01 | 86.86 |  |
| Shigella phage SP18 | GQ981382 | 170605 bp | Linear | *Shigella sonnei* | 0.1 | NA**^*^** |  |
| Shigella phage EP23 | JN984867 | 44077 bp | Linear | *Shigella sonnei* and  *Escherichia coli* | 0.1 | NA |  |
| Shigella phage SSP1 | KY963424 | 113299 bp | Linear | NA | NA | NA |  |
| Shigella phage SFN6B | KY684082 | 43036 bp | Linear | *Shigella flexneri* | NA | NA |  |
| Shigella phage Sf20 | MF327006 | 163982 bp | Linear | NA | NA | NA |  |
| Shigella phage Sf21 | MF327007 | 166002 bp | Linear | NA | NA | NA |  |
| Shigella phage Sf25 | MF327009 | 168573 bp | Linear | NA | NA | NA |  |
| Shigella phage Sf24 | MF327008 | 168112 bp | Linear | NA | NA | NA |  |
| Shigella phage Sf17 | MF327004 | 90092 bp | Linear | NA | NA | NA |  |
| Shigella phage Sf19 | MF327005 | 90375 bp | Linear | NA | NA | NA |  |
| Shigella phage SHSML-45 | KX130863 | 108050 bp | Linear | NA | NA | NA |  |
| Shigella phage Sf14 | MF327003 | 87575 bp | Linear | NA | NA | NA |  |
| Shigella phage pSs-1 | KM501444 | 164999 bp | Circular | *Shigella flexneri,*  *Shigella sonnei* | NA | NA |  |
| Shigella phage Sf23 | MF158046 | 167678 bp | Linear | NA | NA | NA |  |
| Shigella phage Sf22 | MF158045 | 166283 bp | Linear | NA | NA | NA |  |
| Shigella phage Sf18 | MF158044 | 90270 bp | Linear | NA | NA | NA |  |
| Shigella phage Sf16 | MF158043 | 88580 bp | Linear | NA | NA | NA |  |
| Shigella phage Sd1 | MF158042 | 48262 bp | Linear | NA | NA | NA |  |
| Shigella phage Sf15 | MF158041 | 88474 bp | Linear | NA | NA | NA |  |
| Shigella phage Sf13 | MF158040 | 87570 bp | Linear | NA | NA | NA |  |
| Shigella phage Sf12 | MF158039 | 47647 bp | Linear | NA | NA | NA |  |
| Shigella phage Sf11 | MF158038 | 46454 bp | Linear | NA | NA | NA |  |
| Shigella phage SHSML-52-1 | KX130865 | 169621 bp | Linear | NA | NA | NA |  |
| Shigella phage SHBML-50-1 | KX130864 | 166634 bp | Linear | NA | NA | NA |  |
| Shigella phage SHFML-26 | KX130862 | 168993 bp | Linear | NA | NA | NA |  |
| Shigella phage SHFML-11 | KX130861 | 170650 bp | Linear | NA | NA | NA |  |
| Shigella phage Ss-VASD | KR781488 | 62851 bp | Linear | *Shigella sonnei* | NA | NA |  |
| Shigella phage 75/02 Stx | KF766125 | 60875 bp | Circular | *Shigella sonnei* | NA | NA |  |
| Shigella phage Shf125875 | KM407600 | 169062 bp | Linear | NA | NA | NA |  |
| Shigella phage POCJ13 | KJ603229 | 62699 bp | Linear | *Shigella flexneri* | NA | NA |  |
| Shigella phage SfIV | KC814930 | 39758 bp | Linear | *Shigella flexneri* | NA | NA |  |
| Shigella phage SfII | KC736978 | 41475 bp | Linear | *Shigella flexneri* | NA | NA |  |
| Shigella phage phiSboM-AG3 | FJ373894 | 158006 bp | Linear | *Shigella boydii*  *Shigella flexneri* | NA | 152 |  |
| Shigella phage Shfl2 | HM035025 | 165919 bp | Circular | NA | NA | NA |  |
| Shigella phage Shfl1 | HM035024 | 50661 bp | Circular | NA | NA | NA |  |
|  |  |  |  |  |  |  |  |
