# Supplementary material for: Genomic and Proteomic Characterizations of Sfin-1, a Novel Lytic Phage Infecting Multidrug-Resistant Shigella spp. and Escherichia coli C
Source: Front Microbiol. 2019 Aug 22;10:1876. doi: 10.3389/fmicb.2019.01876 (PMC6714547; doi:10.3389/fmicb.2019.01876)
Supplement: TABLE S2 — Comparative analysis of Sfin-1 with pSf-2 and Shfl1 genomes. [file Table_2.DOCX]

**Supplementary TABLE S2**

| **Phages** | **Genome size (nt)** | **GC Content** | **No. of CDS** | **No. of transcription terminator** | **Origin of replication (nt)** |
| --- | --- | --- | --- | --- | --- |
| ***Sfin-1*** | 50,403 | 45.2 | 82 | 43 | 9401 |
| **PSf-2** | 50,109 | 45.4 | 82 | 52 | 11701 |
| **Shfl1** | 50,661 | 45.4 | 83 | 48 | 1 |
